# Uterine microbial communities and their potential role in the regulation of epithelium cell cycle and apoptosis in aged hens

**DOI:** 10.1186/s40168-023-01707-7

**Published:** 2023-11-11

**Authors:** Dong Dai, Jing Wang, Haijun Zhang, Shugeng Wu, Guanghai Qi

**Affiliations:** Laboratory of Quality and Safety Risk Assessment for Animal Products On Feed Hazards (Beijing) of the Ministry of Agriculture and Rural Affairs, Institute of Feed Research, Chinese Academy of Agricultural Sciences, No. 12 Zhongguancun South St., Haidian district, Beijing, 100081 China

**Keywords:** Microbiome, Uterus, Aging, Apoptosis, Microbial metabolite, Hen

## Abstract

**Background:**

Alterations of the uterine microbiome are closely associated with various intrauterine diseases and physiological conditions, which are well-established in mammals. However, as representative oviparous animals, the research on the uterine microbial ecosystem and its functions with physiological homeostasis is limited in chickens. Additionally, continuous egg-laying disrupts the oviducal immune defenses of aged hens, susceptible to pathogen invasion, causing poor egg quality and food-borne infections in humans. Here, we investigated aging-related changes in the oviduct microbial colonization and transmission from the gut to eggs and their roles in a hen model.

**Results:**

The results of 16S rDNA sequencing showed significant differences in the oviduct microbial composition between young (38 weeks) and aged (77 weeks) laying hens. SourceTracker analysis further revealed differences in the effects of microbial transmission on the oviducal microbiota between young and aged hens. Enhanced barrier defense with cell apoptosis suppression and cell cycle arrest of the uterus were observed in aged hens reducing microbial transmission from the lower to upper reproductive tract. In addition, a total of 361 significantly differential metabolites were identified using metabolomics in the aged uterine microbiota, especially in products of amino acid metabolism and biosynthesis of various secondary metabolites, which might have essential effects on cell apoptosis by regulating immune responses and cell cycle. Notably, antibiotics disrupted uterine microbiota by dietary intervention and direct perfusion did not retard aging-related physiological changes but further aggravated aging processes by disrupting the cell cycle and apoptosis.

**Conclusions:**

The microbiota continuum along the reproductive tract in aged birds differs from that in young birds, especially with a significant shift in the uterus. The aged uterine microbiota probably contributes to the regulation of cell cycle and apoptosis by microbial metabolites primarily involved in amino acid metabolism and biosynthesis of various secondary metabolites. These findings provide new insights into the roles of the reproductive tract microbiota in regulating the cell programming of the aged host, contributing to the exploration of the microbiome as a target for diagnosing aging health status and therapy for gynecological diseases in women.

Video Abstract

**Supplementary Information:**

The online version contains supplementary material available at 10.1186/s40168-023-01707-7.

## Background

The emerging field of research focused on the female reproductive tract microbiome reveals a site-specific microbiota in the uterus that plays relevant roles in host gynecological and reproductive health [1–4]. It has been well established that the alteration of the uterine microbiome is closely associated with various intrauterine diseases and different physiological conditions in humans, mice, and bovine [5–7]. However, the research on the uterine microbial ecosystem and its functional relationship with physiological homeostasis is limited in other animals.

As representative oviparous animals, chickens (*Gallus gallus*) have a long tubular oviduct responsible for egg formation and contribute to covering the needs of protein for humans. Microbial contamination of the egg caused by pathogens invading the upper reproductive tract of hens, such as *Salmonella*, leads to food-borne infection outbreaks and seriously endangers public health worldwide [8, 9]. Besides, more influences are exerted by the microbiota of the reproductive tract on egg production than by the digestive tract microbiota of laying hens [10]. The potential for the uterine microbiota to affect health and egg safety has particular relevance for aged hens because long-term and continuous egg-laying disrupts the oviducal immune defense [11, 12]. Uterine inflammatory responses, functional degeneration, and cell senescence that accompany aging are inevitable, which can consequently cause shifts in microbial colonization [13–15]. We hypothesized there might be an adaptive interaction between uterine microbial colonization and age-related changes in the microenvironment. Further studies are needed for a better understanding of the implications of the composition and functional potential of the uterine microbiota in relation to aging and cellular senescence.

In addition, microbial ascension transmission could be another crucial factor in forming distinct microbial ecosystems in the upper reproductive tract. Studies in humans, mammals, and poultry consistently identified a microbiota continuum from the lower to upper reproductive tract that gradually changes [16–19], although female reproductive tracts are quite divergent in both anatomy and physiology among species. While synchronous variation of the uterine and vaginal microbiome with advancing age has been revealed in humans [5, 20], the degree to which microbial transmission from the lower reproductive tract contributes to the aged microbial community in the uterus is uncertain.

In the present study, we aimed to investigate the aging-related changes in the oviduct microbial colonization and transmission from the gut to eggs in a hen model. Subsequently, integrated multi-omics analyses, including metabolomics and transcriptomics, were applied to explore the interactions between the oviducal microbiome and the aged microenvironment. In addition, the aged oviducal microbiota was disrupted by dietary intervention and direct perfusion to verify microbial roles. Collectively, our work reveals the roles of the oviducal microbiota in the regulation of host aging processes, which will not only fill the research gap in poultry but also provide new insights into future prevention and treatment strategies for reproductive tract diseases in women.

## Methods

### Animals

All animal protocols were approved by the Animal Care and Use Committee of the Institute of Feed Research of the Chinese Academy of Agricultural Sciences (approval number: AEC-CAAS-20200902). In this study, a total of 90 37-week-old (peak egg-laying period) and 180 64-week-old (late egg-laying period) healthy Hy-Line Brown hens that were close to average egg production and average eggshell quality were selected respectively from two flocks of 2000 hens each to ensure more representative samples. The experiment was designed as a randomized block. Furthermore, each group was subdivided into 10 replicates with 9 hens each. All hens were housed in three-tier battery cages with three hens per cage (cage size 45 cm × 45 cm × 45 cm) and had free access to feed and water in an environmentally controlled house. Room temperature was maintained at 24°C and provided a controlled photoperiod cycle of 16 h light (lights on at 05:00 AM) per day. Before the initiation of the experiment, all hens were fed the same corn-soybean meal basal diet (Supplementary Table S1) formulated according to National Research Council (1994) recommendations and adapted for 1 week. After 12 weeks of feeding the basal diet for the late egg-laying period group, 77-week-old hens were used as an aged model to investigate phenotypic differences compared to 38-week-old young hens. In addition, the basal diet and basal diet supplemented with 200 mg/kg neomycin (Best Biotechnology Co., Ltd., Hefei, China) were fed for another 12 weeks to verify the role of microbial colonization in the uterine microenvironment of aged hens by the dietary intervention strategy.

### Sample collection

At the end of the feeding trial, one bird close to the average egg production and egg quality was randomly selected from each replicate to be euthanized via cervical dislocation. Furthermore, all sample collection operations were aseptically performed on a clean bench using 75% ethanol to prevent sample contamination, as described by Wen et al. [18]. In detail, microbial samples of the reproductive tract, including the cloaca, vagina, uterus, and magnum, were swabbed using CLASSIQ swabs (Coppan, CA, USA). Additionally, they were suspended in a frozen pipe (Corning, NY, USA) with 2 mL phosphate-buffered saline (PBS). Samples from the eggshell surface and surroundings, including the cages and nipple drinkers, were also collected using swabs. Liquid whole egg, feed, and cecal samples were collected directly for microbial detection. The entire inner surface of the dissected uterus was flushed with 5 mL of deionized water. Furthermore, the recovered fluid was stored in a frozen pipe (Corning, NY, USA). The oviducal mucosa of the uterus was scraped off using a sterile glass slide. These collected samples were frozen in liquid nitrogen and stored at – 80°C until further use.

### Deoxyribonucleic acid (DNA) extraction and 16S rDNA sequencing

Microbial DNA was extracted from a total of 200 samples using the E. Z. N. A. Soil DNA Kit (Omega Bio-Tek, Norcross, GA, USA) according to the manufacturer’s instructions. The quality of the DNA extract was assessed by 1% agarose gel electrophoresis using a NanoDrop 2000 (Thermo Scientific, Wilmington, DE, USA). The hypervariable region V4 of the bacterial 16S rDNA was amplified using forward primer 515F (5′-GTGCCAGCMGCCGCGGTAA-3′) and reverse primer 806R (5′-GGACTACHVGGGTWTCTAAT-3′) [18]. Polymerase chain reaction (PCR) amplification of the 16S rDNA was performed using an ABI GeneAmp® 9700 PCR thermocycler (Applied Biosystems, CA, USA) as follows: 95°C for 3 min, followed by 27 cycles at 95°C for 30 s, 55°C for 30 s, and 72°C for 45 s, and a final step at 72°C for 10 min. Purified 16S rDNA amplicons were pooled in equimolar amounts and sequenced using the Illumina MiSeq platform (Illumina, San Diego, CA, USA). Notably, blank and negative controls were used to exclude potential bacterial contamination during sample measurements in the current study.

Raw fastq files were demultiplexed and quality-filtered using Quantitative Insights into Microbial Ecology 2 [21]. As previously described, amplicon sequence variants (ASVs) were denoised and joined with DADA2 [22]. ASVs were taxonomically classified by using the classify-sklearn Naive Bayes taxonomy classifier [23] against the SILVA (v.138) database [24]. The categorical confidence level was set to 0.7. The Shannon index of the microbiota was estimated using the Mothur software (version 1.30.2). The Bray–Curtis distance was used in the principal coordinate analysis (PCoA), accompanied by the permutational multivariate analysis of variance (PERMANOVA), to calculate statistical significance. Microbial biomarkers were identified using linear discriminant analysis combined effect size (LDA > 4, *P* < 0.05). Spearman’s correlation analysis was used to construct the relationship between intestinal microbiota and phenotypes using the heatmap package. SourceTracker analysis was used to investigate the microbial source and transmission of the oviduct, as previously described by Knights et al. [25].

### Uterine histomorphology analysis

Uterine tissues were fixed with 4% paraformaldehyde solution for 24 h and then embedded in paraffin. Paraffin samples were continuously cut into 5-μm-thick sections.

using a microtome (Leica RM2016, Shanghai, China) and stained with hematoxylin and eosin. The mucosal epithelial thickness and pathological analysis were assessed using a light microscope coupled with the Medical Image Analysis software (Inverted microscope: NIKON CI-S, Tokyo, Japan; Imaging system: NIKON DS-U3, Tokyo, Japan).

### Enzyme-linked immunosorbent assay

Uterine tissue homogenates were prepared in ice-cold PBS and centrifuged at 1000 rpm at 4°C for 20 min. The supernatant was collected to detect the concentration of Interleukin-1β (IL-1β), Interleukin-6 (IL-6), tumor necrosis factor-α, lysozyme, Mucin-2 (MUC2), ovotransferrin, Immunoglobulin A (IgA), Immunoglobulin G (IgG), Immunoglobulin M (IgM), and B cell lymphoma-2 (Bcl-2) using a chicken enzyme-linked immunosorbent assay kit (Shanghai Enzyme-linked Biotechnology Co., Ltd., Shanghai, China) following the manufacturer’s instructions. A Microplate Reader (Bio-Rad, Hercules, CA, USA) measured optical density at 450 nm. In addition, a bicinchoninic acid protein assay kit (Nanjing Jiancheng Bioengineering Institute, Nanjing, China) was used to quantify protein concentrations.

### Antibiotic intervention for oviduct microbiota

Neomycin (Thermo Fisher Scientific) was administered via oviduct perfusion (dose 0.5%; volume 2.0 mL) and hens were treated daily for seven days. In detail, the antibiotics were freshly prepared before perfusion and using sterile PBS to dissolve the powder. The cloaca was wiped with 75% ethanol in a supine position for hens. It was then softly pressed into the oviductal opening following artificial insemination. Next, a disposable sterile inseminating tube was used to instill neomycin solution into the uterus slowly. Finally, the hen was kept in an upside-down position for 5 min and then returned to the cage. Notably, hens tolerated the antibiotic treatment well, and no stress behaviors or diarrhea were observed compared to the control group.

### Egg quality analysis

Eggs were collected to measure eggshell strength, thickness, and color. Eggshell strength and shell thickness were detected using the Egg Force Reader (Israel Orka Food Technology Ltd., Ramat Hasharon, Israel) and Eggshell Thickness Gauge (Israel Orka Food Technology Ltd., Ramat Hasharon, Israel), respectively. Eggshell color was evaluated based on the L, a, and b values using a colorimeter (NH310; 3nh Co., Shenzhen, China).

### Quantification of bacterial load

The copy number of the bacterial 16S rRNA gene was determined by the absolute quantitative PCR method. The primers Eub338 (5′-ACTCCTACGGGAGGCAGCAG-3′) and Eub518 (5′-ATTACCGCGGCTGCTGG-3′) were used for amplification of the bacterial 16S rRNA gene. Firstly, recombinant plasmids were constructed. In brief, amplification of the target gene was performed by PCR, and the amplified product was recovered and purified. They were then connected to the cloning vector PMD18-T and transformed with the competent *E. coli*. After extraction of recombinant plasmids, sequencing was performed to ensure the exact orientation and position of the inserted fragments. Next, the standard curve was constructed. The OD260 of recombinant plasmids was determined using a NanoDrop2000 spectrophotometer (Thermo Fisher Scientific, DE, USA) and converted to the number of copies. Then, plasmids were serially tenfold diluted and used to construct standard curves. Finally, after amplification of the target gene in the sample using an ABI7300 fluorescence quantitative PCR instrument (Applied Biosystems, Foster City, CA, USA), the number of bacterial DNA copies was calculated based on the constructed standard curve.

### Metabolome analysis

In total, 100 μL of flush fluid (10 samples/group) from the uterus was extracted using 400 µL of methanol: acetonitrile (1:1, v/v) solution. After sonication at 40 kHz for 30 min at 5°C, the samples were treated at -20°C for 30 min to precipitate proteins. The cleared supernatant was carefully transferred to sample vials for liquid chromatography-mass spectrometry analysis after centrifugation for 15 min at 13000 × *g* at 4°C. Equal volumes of all samples were mixed to prepare a quality control sample. Liquid chromatography-mass spectrometry analysis was performed using an ultra-high performance liquid chromatography-quadrupole (UHPLC-Q) system equipped with an HSS T3 column (100 mm × 2.1 mm i.d., 1.8 µm; Waters, Milford, USA). A Thermo UHPLC-Q Exactive Mass Spectrometer equipped with an Electrospray Ionization source operating in either positive or negative ion mode was used to collect mass spectrometric data. Finally, the raw data were imported into Progenesis QI 2.3 (Nonlinear Dynamics, Waters, USA) for peak detection, correction, and alignment to generate a data matrix including retention time, mass-to-charge ratio values, and peak intensity.

The data matrix was preprocessed using the Majorbio platform for missing value recording and normalization. In detail, variables with more than 80% of metabolic features were retained in any set of samples. To reduce the errors caused by sample preparation and instrument instability, the response intensities of the sample mass spectrum peaks were normalized by the sum-normalization method to obtain the normalized data matrix. In addition, the variable with a relative standard deviation > 30% in the QC samples was excluded. To obtain the final data matrix for subsequent analysis, log10 processing was performed. The metabolites were matched against the database based on Kyoto Encyclopedia of Genes and Genomes (KEGG) (http://www.genome.jp/kegg/) and the Human Metabolome Database (http://www.hmdb.ca/) to annotate information on metabolites. The variable importance in the projection (VIP) obtained by the Orthogonal Projections to Latent Structures Discriminant Analysis model and the *P* value obtained by Student’s* t*-test were used to determine significantly differential metabolites (VIP > 1 and* P* < 0.05). In addition, KEGG pathway enrichment analysis was performed using the Python package (scipy.stats, https://docs.scipy.org/doc/scipy/) and Fisher’s exact test. Interactive pathway analysis using iPath3.0 was conducted as described previously [26].

### RNA-sequencing (RNA-Seq) transcriptome analysis

Total RNA was extracted from uterine samples of young and aged hens (10 samples/group) using TRIzol Reagent following the manufacturer’s instructions, and genomic DNA contamination was removed using DNase I to obtain purified total RNA. Ribonucleic acid (1 μg) was used to prepare the RNA-seq transcriptome library using the TruSeqTM RNA sample preparation Kit from Illumina (San Diego, CA, USA). An Illumina NovaSeq 6000 sequencer (Illumina, CA, USA) was used to sequence the paired-end RNA-seq sequencing library. Fastp [27] and hierarchical indexing for spliced alignment of transcripts 2 [28] were used to trim and quality control the raw paired-end reads and align the clean reads reference genome separately. StringTie was used to assemble the mapped reads of each sample [29]. The expression level of each gene was calculated using RSEM according to the fragments per kilobase per million reads method [30]. Differential expression analysis was performed using DESeq2 [31]. Genes with |log_2_fold change|> 1 and *P* adjust < 0.05 were considered to be differentially expressed genes (DEGs). The DEGs clustering analysis was performed based on the k-mean algorithm [32]. In detail, K initial cluster centroids were randomly selected and then the distance between each gene and each cluster centroid was calculated based on the Euclidean distance algorithm. Each gene was assigned to the cluster centroid that was closest to it. The cluster centroids and the genes assigned to them represent a cluster. For each gene assigned, the cluster centroids were recalculated based on the existing genes in the cluster. This process was repeated until no more cluster centroids were changed. Functional enrichment analysis was performed using the Gene Ontology (GO) database (http://geneontology.org/) and the KEGG database. Topology-based GO scoring was used to conduct GO functional enrichment analysis [33] and Benjamini–Hochberg corrected* P* values to obtain a false discovery rate (FDR).

### Quantitative real-time PCR (RT-PCR) assay

Real-time PCR analysis was performed to determine gene expression as previously described [34]. Total RNA was isolated from the jejunal mucosa using TRIzol reagent (Tiangen Biotech Co., Ltd, Beijing, China) following the manufacturer’s instructions. RNA concentration was measured using a NanoDrop spectrophotometer (NanoDrop Technologies, Wilmington, DE, USA). The cDNA was obtained by reverse transcription reactions of the total RNA using the First-Strand cDNA Synthesis SuperMix (TransGen Biotech Co., Ltd., Beijing, China). The Applied Biosystems 7500 real-time PCR System with PowerUpTM SYBRTM Green Master Mix (Thermo Fisher Scientific, Waltham, MA, USA) was used to conduct RT-PCR. Relative mRNA expression levels were normalized to those of β-actin using the 2^−ΔΔCt^ method [35]. Primer sequences used in this study are listed in Supplementary Table S2.

### Flow cytometric analysis of cell cycle

Freshly dissected uterine tissue was cut into small pieces of less than 1 mm with scissors and repeatedly washed with phosphate-buffered saline. The tissue blocks were transferred to 1 mg/mL collagenase IV and digested in a water bath kettle at 37 °C for 70 min. The digestion solution was filtered through a 300-mum nylon mesh. Furthermore, the filtrate was centrifuged at 600 rpm for 10 min, and the supernatant was discarded. The cells were slowly added to 70% precooled ethanol and stored at 4 °C overnight. After washing with cold phosphate-buffered saline, the cells were resuspended in 1 mL staining reagent (50 mg/mL PI, 100 mg/mL RNase). The cells were incubated on ice for 30 min in the dark. A minimum of 1 × 10^6^ cells per sample were collected for cell cycle analysis using a Coulter Epics XL flow cytometer (Beckman Coulter, CA, USA).

### Terminal deoxynucleotidyl transferase-mediated dUTP nick end labeling (TUNEL) assay

Apoptosis of uterine tissues was detected by the TUNEL assay in hens using a fluorescein TUNEL cell apoptosis detection kit (Wuhan Servicebio Technology Co., Ltd., Hubei, China). Slides were prepared for histomorphological analysis. Hydrogen peroxide (3%) was used to block the endogenous peroxidase activity. Then the slides were incubated with recombinant TdT enzyme diluent for 1 h at 37 °C and were rinsed with PBS four times for 5 min each. The slides were dipped in a PI solution in the dark for 8 min at room temperature. After rinsing with PBS four times, the slides were dripped in an antifade mounting medium. Finally, the slides were examined under a fluorescence microscope, and Image Pro Plus 6.0 was performed for image analysis.

### Statistics

Statistical Analysis Software Version 9.2 (SAS Institute Inc., Cary, NC, USA) was used for statistical analysis unless otherwise indicated. Statistical significance between the two groups was evaluated using a two-tailed Student’s t-test. For microbial statistics, the statistical significance between the two groups was evaluated using the Wilcoxon rank-sum test. Furthermore, the differences among the three groups were analyzed using the Kruskal–Wallis test. Statistical significance was represented by *P* < 0.05, while a tendency toward significance was considered at 0.05 ≤ *P* < 0.10.

## Results

### The uterus of aged hens reduces microbial transmission from the lower to upper reproductive tract

**Fig. 1:**
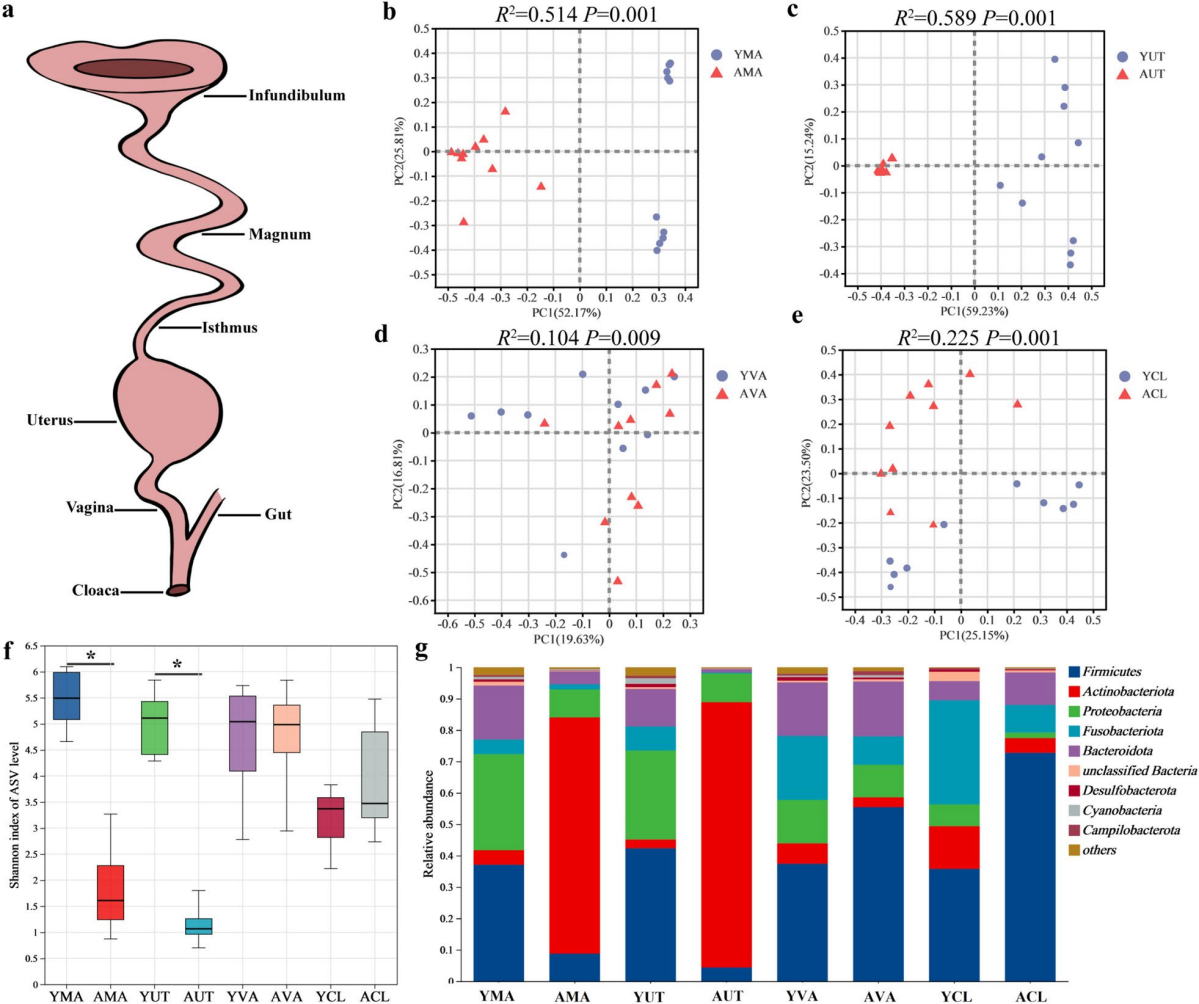
Differences in the microbial composition of the oviduct in young and aged hens (*n* = 10). **a** Diagram of the reproductive tract of chickens. **b**–**e** Principal coordinate analysis (PCoA) of oviduct microbiota based on Bray–Curtis. **f** Comparison of Shannon index of α-diversity. **g** Microbial composition at the phylum level of the oviduct in young and aged hens. YMA, young magnum; AMA, aged magnum; YUT, young uterus; AUT, aged uterus; YVA, young vagina; AVA, Aged vagina; YCL, young cloaca; ACL, aged cloaca

The microbiota in the reproductive tract, including the cloaca, vagina, uterus, and magnum (Fig. 1a), was profiled to elucidate the microbial shifts and explore the potential source and transmission of oviducal microbiota in hens at peak-laying (38 weeks of age) or late-laying period (77 weeks of age). Principal coordinate analysis showed clear segregation of microbial samples from the reproductive tract between young and aged hens (*P* < 0.05, Fig. 1b–e). Prominent changes in microbiota related to age were observed at the upper sites along the reproductive tract, as evidenced by the decreased Shannon index in the aged magnum and uterus (*P* < 0.05, Fig. 1f) and distinct microbial communities in the aged magnum and uterus from that of the young (Fig. 1g). Specifically, *Actinobacteria* was dominant in the aged magnum and uterus. In contrast, *Firmicutes*, *Proteobacteria*, and *Bacteroidetes* were the major phylum in the young magnum and uterus. These results suggest the potential selectivity of microbial colonization in the reproductive tract of aged hens.

**Fig. 2:**
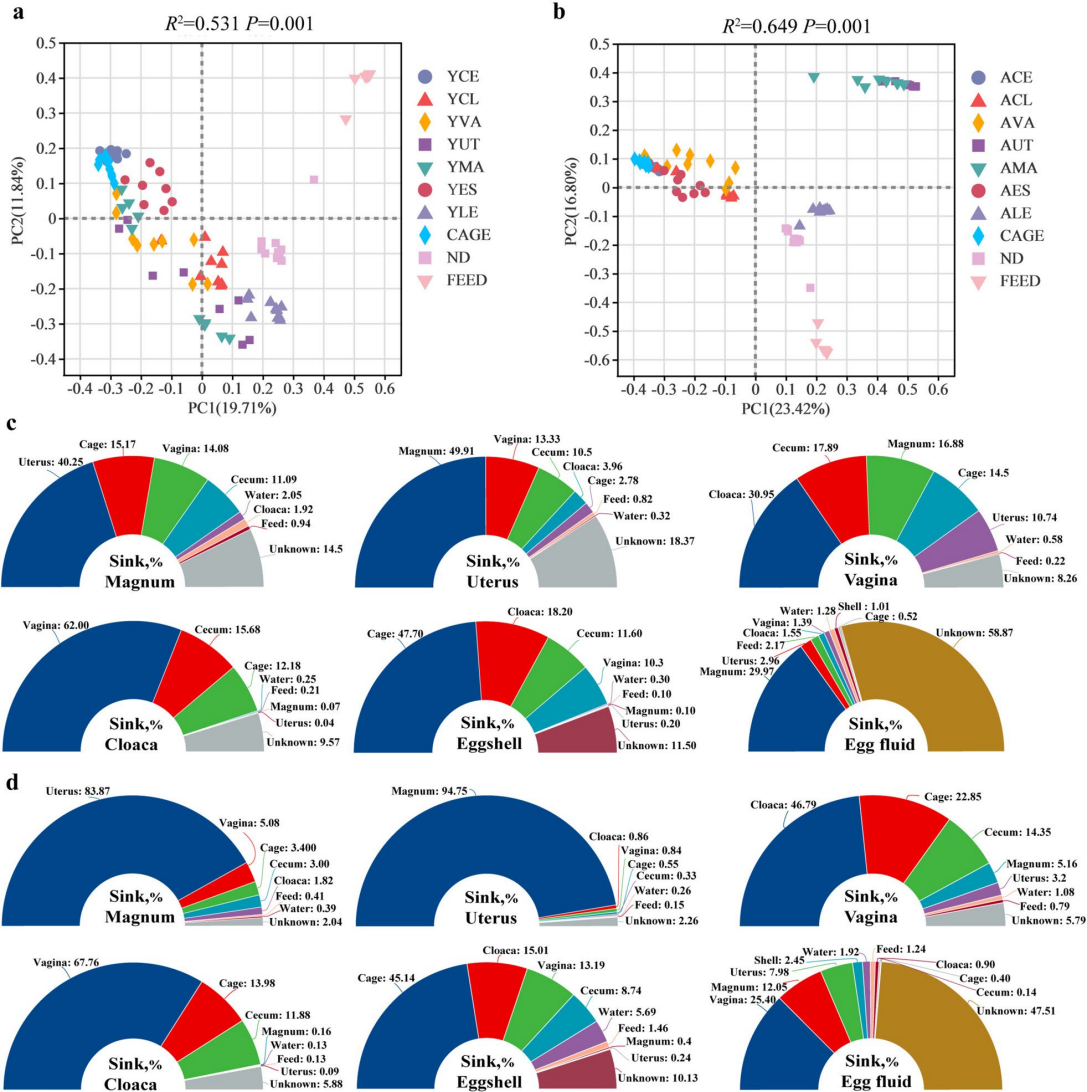
Microbial transmission and SourceTracker analysis in the reproductive tract of hens (*n* = 10). **a** Principal coordinate analysis (PCoA) in the young group based on Bray–Curtis. **b** PCoA in the aged group based on Bray–Curtis. **c** The SourceTracker analysis in the young group. **d** The SourceTracker analysis in the aged group. YMA, young magnum; AMA, aged magnum; YUT, young uterus; AUT, aged uterus; YVA, young vagina; AVA, Aged vagina; YCL, young cloaca; ACL, aged cloaca; YCE, young cecum; YES, young eggshell. YLE, young liquid whole egg; ND, nipple drinkers; ACE, aged cecum; AES, aged eggshell. ALE, aged liquid whole egg

To investigate effects of the microbial transmission on the microbiota in the reproductive tract, we characterized microbial samples from the gut, eggs, and surroundings, including cages, feed, and nipple drinkers to identify microbial profiles by 16S rDNA sequencing. Supplementary Figure S1 shows a relative abundance bar graph of microbial composition for all samples. In the scatter plot of the PCoA analysis, the microbial samples of the young reproductive tract, including the vagina, uterus, and magnum, were aggregated with the intestinal and egg samples (*P* < 0.05, Fig. 2a). In the aged group, cloacal and vaginal samples remained aggregated with cages, intestines, and eggshells (*P* < 0.05). However, samples from the uterus and magnum occupied distinct positions that were separated from other samples (*P* < 0.05, Fig. 2b). To clarify the differences between groups, the sample distances of each two groups were analyzed individually using PERMANOVA (Supplementary Table S3). We further used SourceTracker analysis to verify the PCoA results and reveal differences in the effects of microbial transmission on oviducal microbiota between young and aged hens (Fig. 2c). When the magnum was assigned as the sink in the young group, in total 56.2% the microbiota were obtained from reproductive tract sources (uterus, 40.2%; vagina, 14.1%; cloaca, 1.9%). Cecal and environmental sources contributed 11.1% and 15.2% (cage, 15.2%; feed, 0.9%; water, 2.0%) of the magnum microbiota, respectively. Likewise, 67.2% of microbiota were obtained from reproductive tract sources (magnum, 49.9%; vagina, 13.3%; cloaca, 4.0%), 10.5% from cecal sources, and 3.9% from environmental sources (cage, 2.8%; feed, 0.8%; water, 0.3%) for the uterus. As a result, microbial transmission patterns in the "gut-oviduct-egg" of young hens were determined by assigning the cloaca, vagina, eggshell, and egg fluid as the sink. However, the oviduct microbial colonization of aged hens does not seem to follow this transmission pattern closely as that of young hens (Fig. 2d). The contribution of the uterine microbiota (83.9%) increased in the magnum of aged hens as the sink. When the uterus of aged hens was assigned as the sink, the magnum source contributed 94.7%. In contrast, the vagina contributed only 0.8% of the uterine microbiota. This observation indicates that uterus microbial colonization of aged hens is not vulnerable to vaginal microbes, which highlights the interdependent relationship between the microbiome of the upper and lower reproductive tracts in the normal state. Collectively, these results show that the microbial composition and transmission routes of the reproductive tract in aged birds differ from those in young hens. Furthermore, the uterus of aged hens reduced microbial transmission from the lower to upper reproductive tract, which established a unique microbial community characterized by a high proportion of *Actinobacteria*.

### Aged uterus tends toward enhanced barrier defense with cell apoptosis suppression and cell cycle arrest

**Fig. 3:**
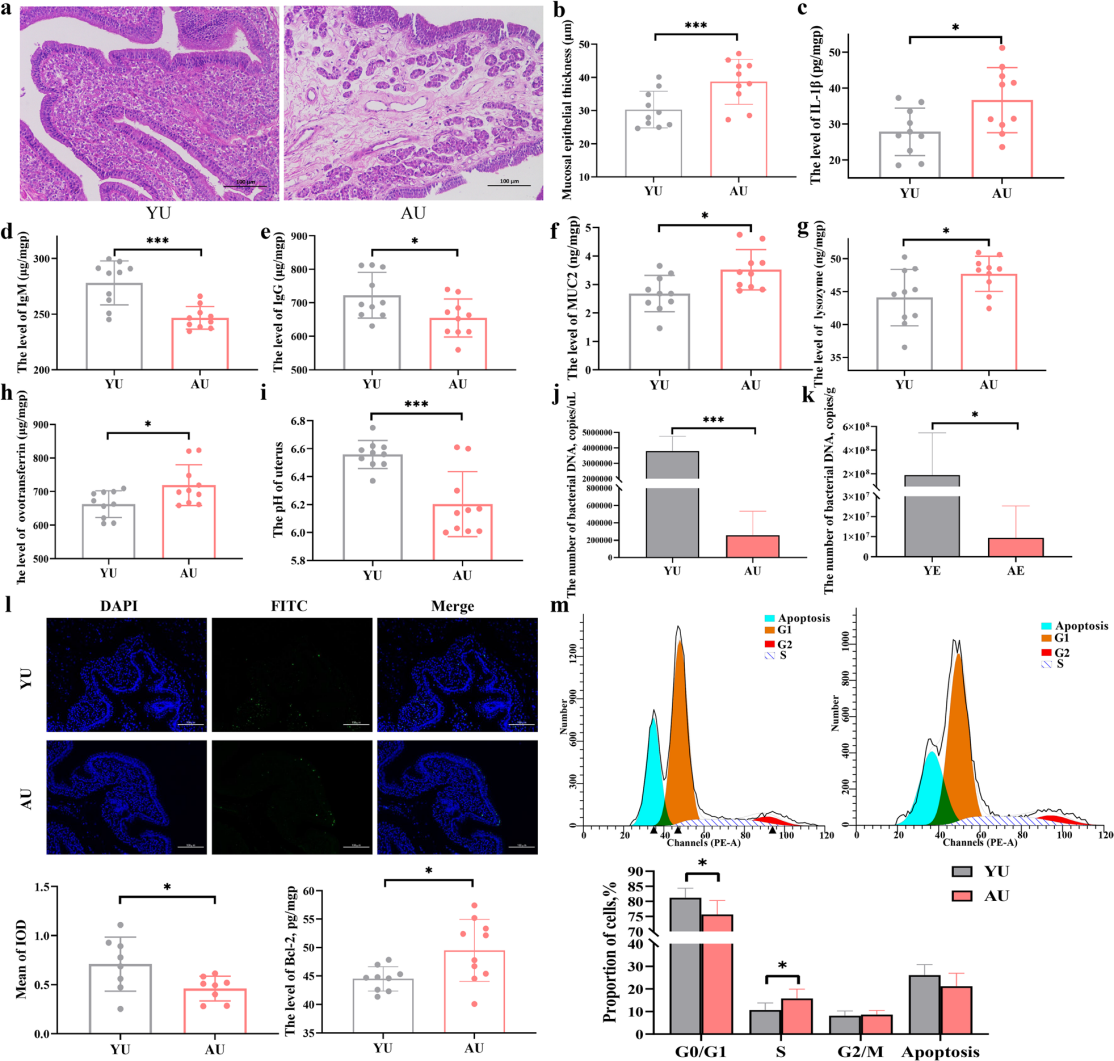
Changes in the microenvironment of the uterus in aged hens. **a** The morphological structure of young and aged uterus. Scale bar = 100 μm. **b**–**i** Comparison of the microenvironment in terms of mucosal epithelial thickness, IL-1β, immunoglobulin, MUC2, lysozyme, ovotransferrin, and pH (*n* = 10). **j** The number of bacterial DNA copies is based on the total DNA (*n* = 10). **k** The number of bacterial DNA copies based on the liquid egg (*n* = 10). **l** Quantitative analysis of apoptotic cells using the TUNEL assay (*n* = 8) and the level of B cell lymphoma-2 (Bcl-2, *n* = 10) in the uterus mucosa. Scale bar = 50 μm. **m** Peak plots for flow cytometry analysis in the uterus of hens (*n* = 10). YU, young uterus; AU, aged uterus; MUC2: Mucin-2; IL-1β: Interleukin-1β; IgG: Immunoglobulin G; IgM: Immunoglobulin M. YE, young liquid whole egg; AE, aged liquid whole egg

Compared with young uterine tissue, signs of edema, loose arrangement of connective tissue, and widening of glandular spacing were observed in the lamina propria of aged uterine tissue, accompanied by inflammatory cell infiltration, increased IL-1β levels, and mucosal epithelial thickness (Fig. 3a-c). Simultaneously, uterine immunoglobulin synthesis was reduced in aged hens, as suggested by the decreased levels of IgG and IgM (Fig. 3d, e). In contrast, aged hens showed higher levels of MUC2 and antibacterial proteins, including lysozyme and ovotransferrin (Fig. 3f–h), in the uterus to strengthen host innate immunity compared to young hens. We found that the pH of the aged uterus was lower than that of the young uterus (Fig. 3i). These changes in the uterus may explain, in large part, the decreased absolute number of bacterial DNA in both the aged uterus and the egg fluid (Fig. 3j, k). Significant correlations were observed, especially in the levels of MUC2, IgA, IgM, lysozyme, and pH, with the relative abundance of uterine microbes (|*R*|> 0.6, *P* < 0.05, Supplementary Figure S2). Furthermore, the results of the TUNEL assay showed a decreased number of positive cells and mean integrated optical density in the aged uterus, suggesting suppression of cell apoptosis in the uterus (Fig. 3l). Meanwhile, a higher level of the anti-apoptotic protein Bcl-2 was also found in the aged uterus than in the young uterus. Flow cytometry observed a reduced proportion of G0/G1-phase cells and an increased proportion of S-phase cells in the aged uterus (Fig. 3m), indicating an increased rate of cell proliferation from the G1 to G2 phase and the presence of S-phase cell cycle arrest in the aged uterus.

**Fig. 4:**
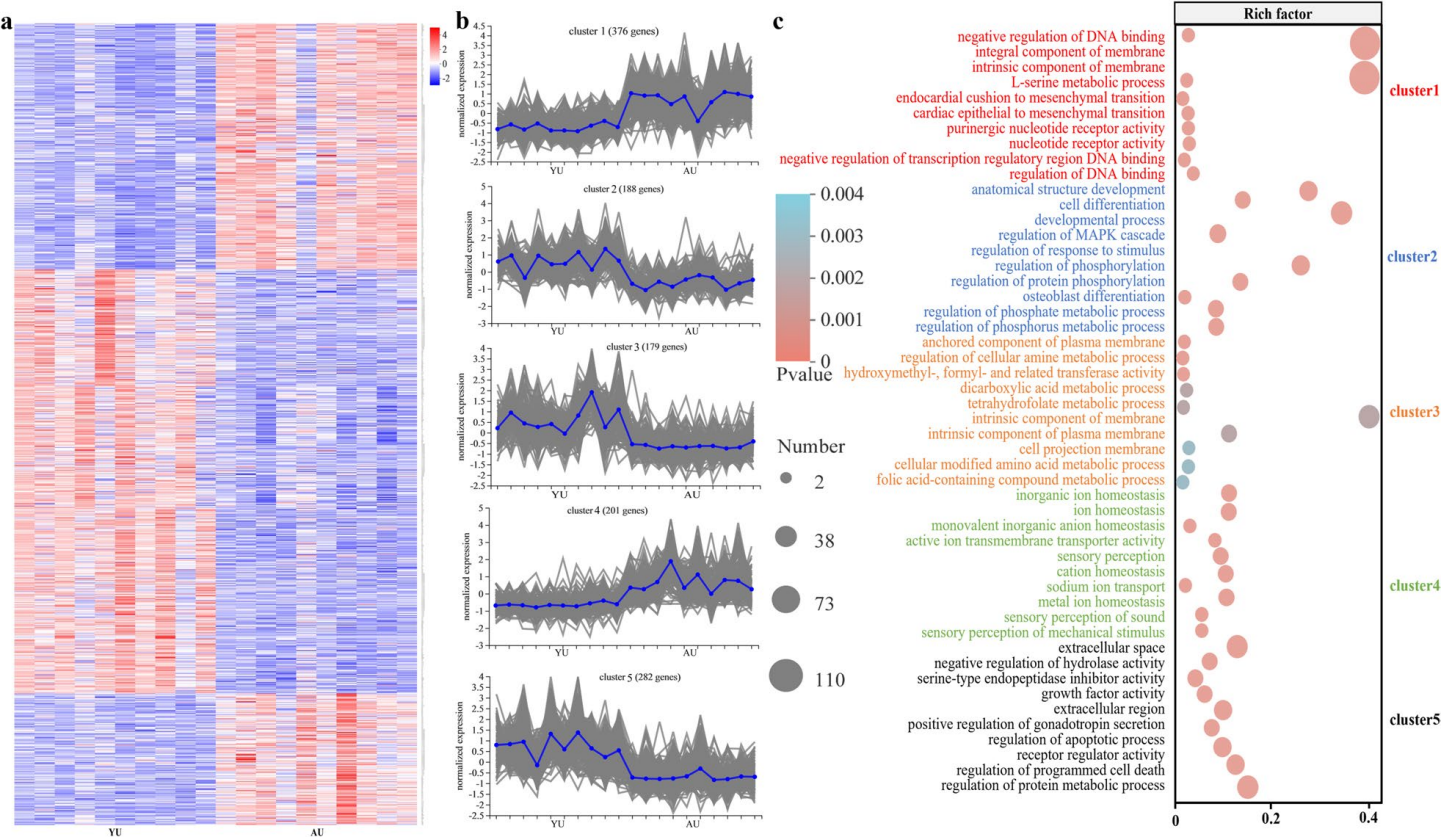
RNA-seq analysis and validation in young and aged hens (*n* = 10). **a** Heatmap of the differential gene clustering. **b** Expression profiles of the five clusters revealed by k-means clustering. **c** Top 10 significantly enriched Gene Ontology (GO) terms in each cluster. YU, young uterus; AU, aged uterus

Gene expression patterns related to aged changes in the uterine epithelium were characterized by the RNA sequencing-based transcriptomic analysis. In total, 1226 DEGs were identified in the uterus (|log_2_fold change|> 1 and *P* adjust < 0.05, Supplementary Figure S3a, b, and Supplementary Table S4). Next, we investigated the primary patterns of changes in gene expression in the aged uterus and identified five clusters using the k-means algorithm (Fig. 4a, b). We found that 376 genes in cluster 1 were typically upregulated in the aged uterus. Gene Ontology enrichment analysis suggested that these genes were primarily involved in transcriptional regulation and DNA binding (Fig. 4c). In contrast, a total of 188 genes in cluster 2 and 282 genes in cluster 5 were typically downregulated in the aged uterus (Fig. 4b). The genes in cluster 2 were primarily involved in cell differentiation, developmental processes, and phosphorus metabolic processes, while genes in cluster 5 were mainly related to the regulation of cell apoptosis and functional activity (Fig. 4c). In addition, the expression of 179 genes in cluster 3 enriched to folic acid-containing compounds and regulation of cellular amine metabolic processes was downregulated in the aged uterus, while genes of cluster 4 enriched to ion transport and homeostasis were typically upregulated (Fig. 4c). The results of the functional annotation of clusters 1 and 5 further confirmed the previously identified changes in cell cycle and apoptosis in the aged uterus. Key genes in each cluster were identified by protein–protein interaction network analysis based on the Search Tool for the Retrieval of Interacting Genes/Proteins database (Supplementary Figure S3c–g). We found that elevated expression of *CCNB1*, *NDC80*, *BUB1B*, *NEK2*, and *CCNB3* accounted for the accelerated cell cycle, while suppressed expression of *FOS*, *BDNF*, *OVAL*, *KLF4*, *AVBD11*, *MMP10*, *CD8A*, *FCER1G*, *AREG*, and *PRKCD* was primarily responsible for the suppression of cell apoptosis. These key genes were selected for RT-PCR verification, and the results were consistent with those of RNA-seq (Supplementary Figure S4). In short, the aged uterus showed histological injury, cell apoptosis suppression, and cell cycle arrest in the epithelium, as well as a higher potential barrier defense against microbes than young hens, which likely contributed to modifying the uterine microbiota.

### Metabolomics analyses reveal the metabolic potential of aged uterine microbiota in the regulation of the host cell cycle and apoptosis

**Fig. 5:**
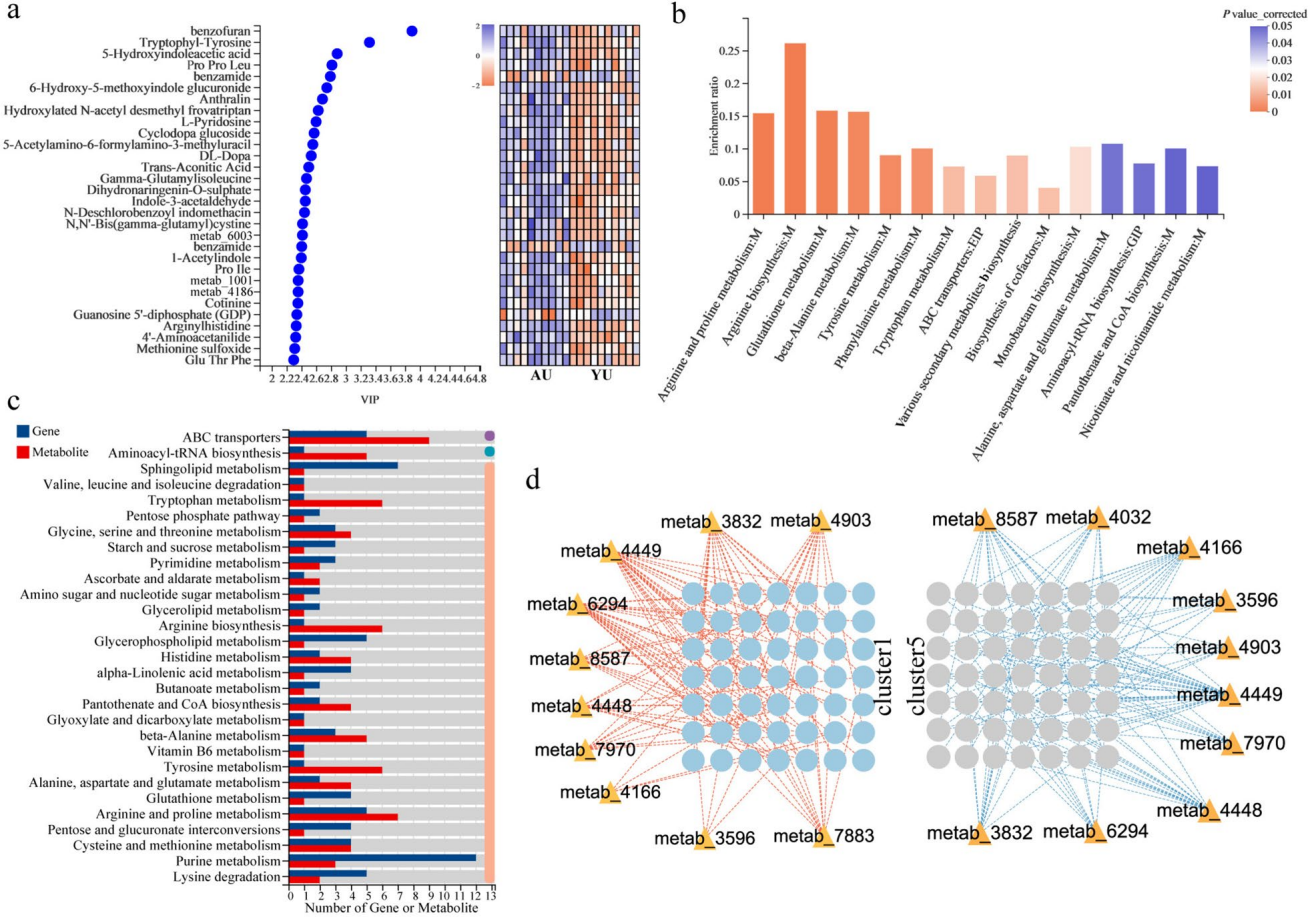
Integrated analysis of metabolomics and transcriptomics in young and aged uterus (*n* = 10). **a** Identification of the importance of differential metabolites for the top 30. **b** Pathway enrichment analysis for upregulated metabolites in aged hens. **c** The shared metabolic pathway between metabolomics and transcriptomics. **d** Spearman’s correlation analysis between differential metabolites and gene sets. |*R*|> 0.80,* P* < 0.05. The red and blue lines represent positive and negative correlations, respectively

To gain further insight into the changes in the microbial metabolites, an untargeted metabolomics analysis was performed using uterine flush fluid of the young and aged uterus. Principal component analysis showed that metabolites of samples were clustered within each group. In total, 361 significantly differential metabolites were identified (*P* < 0.05, VIP > s1, Supplementary Figure S5a, b, and Supplementary Table S5). The impact of these metabolites on the classification discrimination was assessed by analyzing the variable important in projection between the two groups. We found that only the levels of benzamide and guanosine 5'-diphosphate were significantly lower in the aged uterine lavage fluid. However, other metabolites were significantly higher in the top 30 of VIP (Fig. 5a). Further, the pathway enrichment analysis was performed using the Benjamini–Hochberg correction method. We found that in total, 15 metabolic pathways were significantly enriched in the aged uterus, especially in amino acid metabolism and biosynthesis of various secondary metabolites (*P* adjust < 0.05, Fig. 5b). Additionally, furfural degradation, purine metabolism, starch and sucrose metabolism, and ascorbate and aldarate metabolism were significantly enriched in the young uterus (Supplementary Figure S5c).

Next, we explored how the uterine microbiota affects aging-related gene expression patterns. The metabolome and transcriptome were integrated by constructing the O2PLS model with tenfold cross-validation (R2X = 0.806, R2Y = 0.655, Supplementary Figure S6), which suggested significant correlations between them. The differential metabolite set and the differential gene set were annotated in a metabolic overview map using iPath3.0, achieving data integration of the metabolome and transcriptome. We found that amino acid metabolism, energy metabolism, carbohydrate metabolism, nucleotide metabolism, and biosynthesis of other secondary metabolites overlapped and were simultaneously regulated by the microbiota and uterus, especially in various amino acid metabolisms (Supplementary Figure S7, Fig. 5c). Furthermore, a Spearman’s correlation network was constructed based on metabolomics and transcriptomics to explore possible interactions between microbial metabolites and the cell cycle and apoptosis profiles in the uterus (|*R*|> 0.8,* P* < 0.05). A total of 11 metabolites showed a significant positive correlation with the gene sets related to the cell cycle, while a significant negative correlation with the gene sets related to cell apoptosis (Fig. 5d, and Supplementary Table S6). Collectively, these results indicate the potential of aged uterine microbiota to affect host cell apoptosis and cell cycle progression in the epithelium, which might be related to amino acid metabolism and the biosynthesis of various secondary metabolites.

### Microbiota dysbiosis induced by antibiotic disruption inhibits cell apoptosis and aggravates pathological deterioration in the aged uterus

**Fig. 6:**
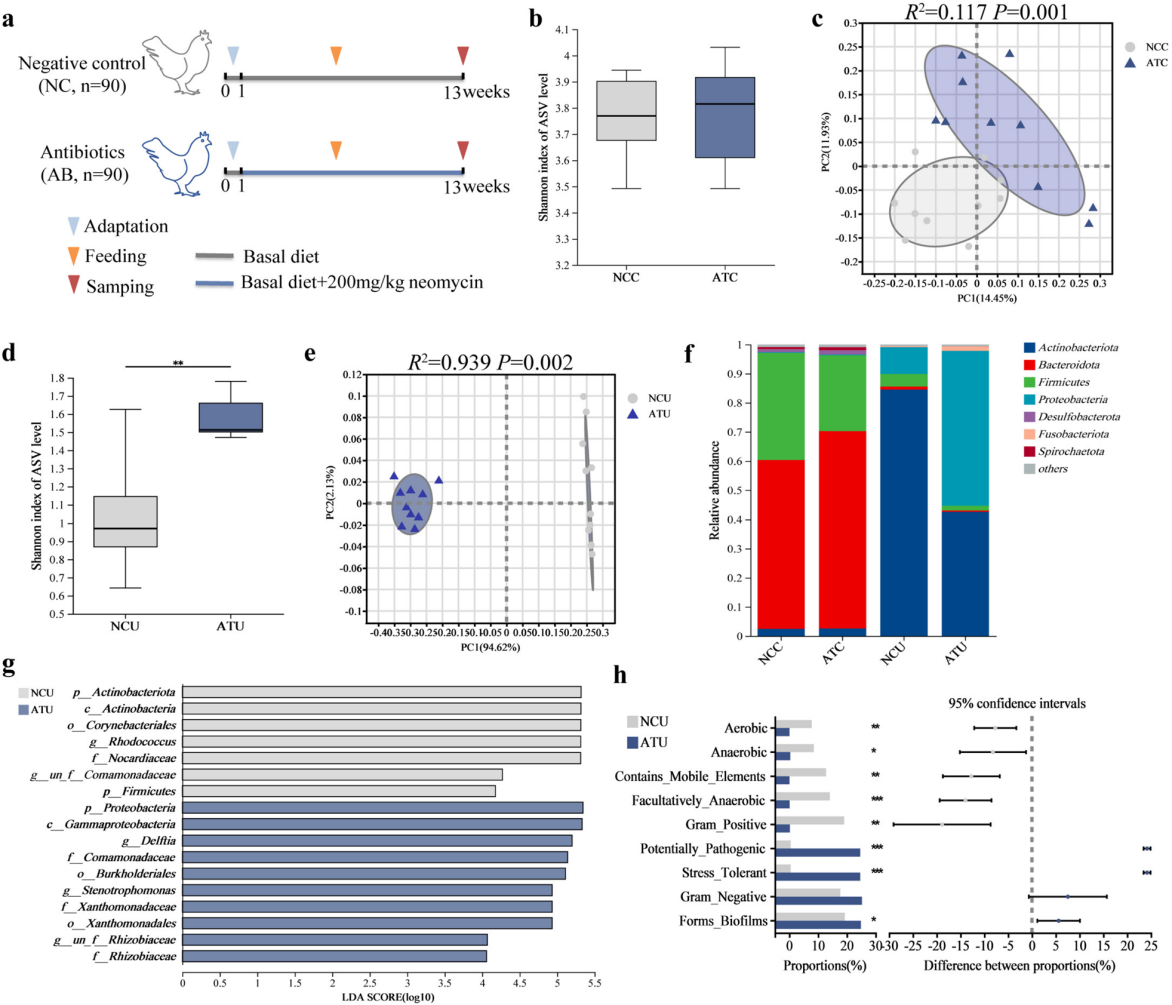
Effects of dietary antibiotic supplementation on cecal and uterine microbiota in aged hens (*n* = 10). **a** Experimental design overview. **b** Comparison of α-diversity in cecal microbiota. **c** Principal coordinate analysis (PCoA) of cecal microbiota based on Bray–Curtis. **d** Comparison of α-diversity in uterine microbiota. **e** PCoA of uterine microbiota based on Bray–Curtis. **f** Microbial composition at the phylum level in cecal and uterine microbiota. **g** Linear discriminant analysis effect size of intestinal microbiota (LDA > 4, *P* < 0.05). **h** The microbial phenotype analysis based on the BugBase database

**Fig. 7:**
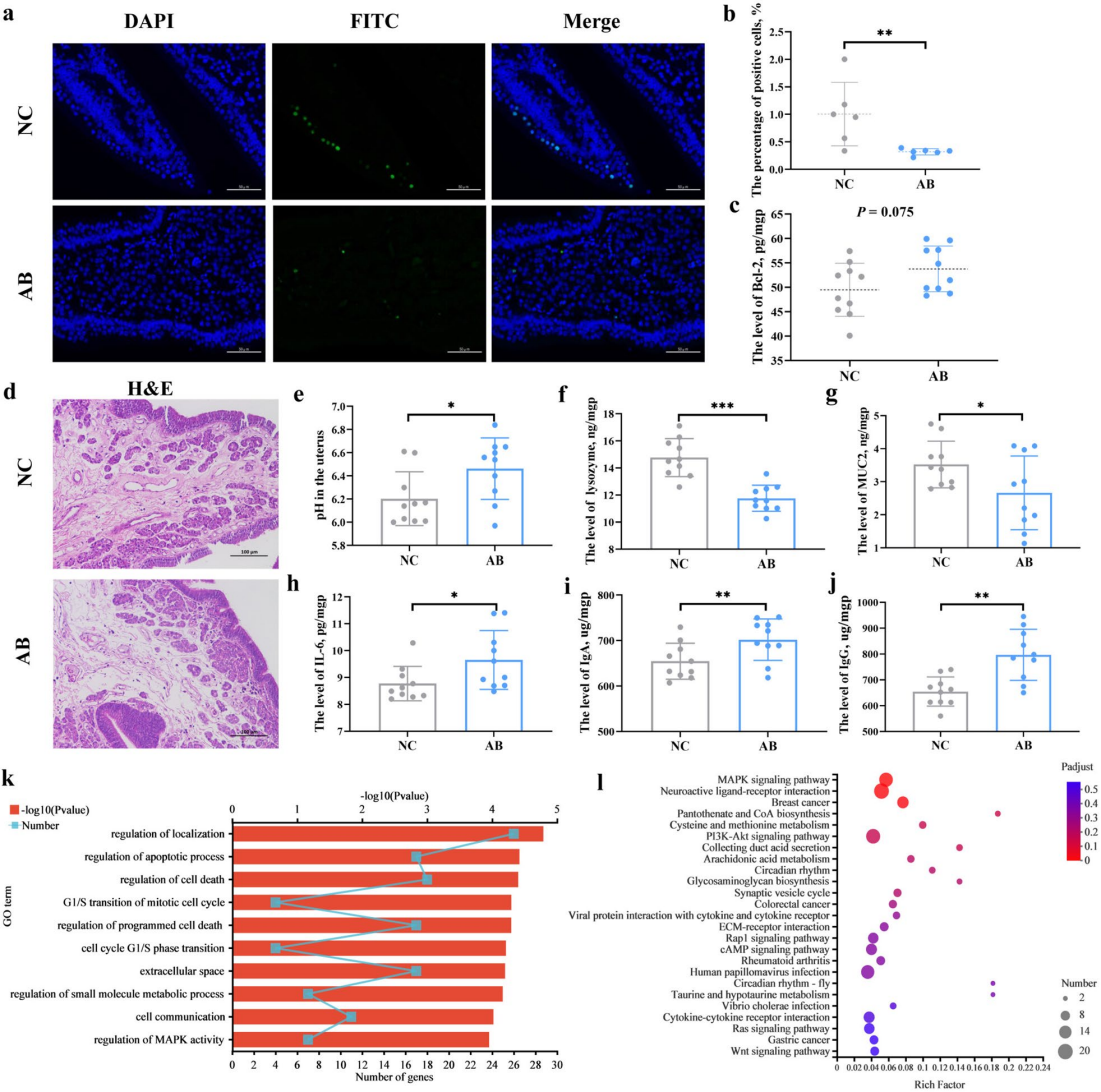
Effects of dietary antibiotic supplementation on uterine microenvironment in aged hens (*n* = 10). **a** Detection of cell apoptosis in uterine tissue using the TUNEL assay. Scale bar = 50 μm. **b** The proportion of cell apoptosis. **c** The level of B cell lymphoma-2 (Bcl-2) in the uterus mucosa. **d** Effects of dietary antibiotic supplementation on the morphological structure of the uterus. Scale bar = 100 μm. **g**–**j** Comparison of the microenvironment in terms of pH, lysozyme, MUC2, IL-6, IgA, and IgG. **k** Significantly enriched Gene Ontology (GO) terms of upregulated genes in the antibiotic group. **l** The Kyoto Encyclopedia of Genes and Genomes (KEGG) enrichment analysis. NC, the negative control group; AB, the antibiotic group; NCC, the negative control cecum; ATC, the antibiotic cecum; NCU, the negative control uterus; ATU, the antibiotic uterus; MUC2: Mucin-2; IL-6: Interleukin-6; IgA: Immunoglobulin A; IgG: Immunoglobulin G

To verify the potential role of microbiota in the host uterine epithelium, the aged uterine microbiota was disturbed by feeding laying hens with neomycin for 12 weeks or direct perfusion with neomycin (Fig. 6a). In the feeding trial, dietary neomycin significantly increased uterine microbial α-diversity in aged hens, but no significant difference was observed in the cecum (Fig. 6b, d). PCoA analysis showed that cecal and uterine samples from the neomycin group were separated from the control group (Fig. 6c, e), suggesting that the microbial community had shifted in response to the antibiotic disruption. In the cecum, the abundances of *Lactobacillus*, *Blautia*, *Romboutsia*, *Turicibacter*, and *Faecalitalea* were depleted by dietary neomycin (Supplementary Figure S8a). In the uterus, the abundance of *Actinobacteriota*, *Firmicutes*, *Rhodococcus*, and *unclassified_f_Comamonadaceae* was significantly decreased. *Proteobacteria*, *Delftia*, *Stenotrophomonas*, and *unclassified_f_Rhizobiaceae* were identified as the biomarkers in the uterus (LDA > 4, Fig. 6g). Moreover, the phenotype analysis revealed significant changes in the uterus by antibiotic disruption based on the BugBase database (Fig. 6h). Accompanied by microbiota dysbiosis, antibiotic intervention tended to increase the level of the anti-apoptotic protein Bcl-2, consequently suppressing cell apoptosis of the uterus in aged hens (Fig. 7a–c). In addition, we found that neomycin aggravated the pathological condition of the uterus in aged hens, including signs of edema, loose arrangement of connective tissue, and widening of glandular spacing (Fig. 7d). Simultaneously, significant changes in pH, antibacterial properties, and inflammation induced by dietary antibiotic interventions were observed in the aged uterus (Fig. 7e–j). Dietary neomycin significantly increased eggshell thickness and reduced eggshell color (Supplementary Figure S9). These results indicate that antibiotics caused dysbiosis of the uterine microbiota, inhibited epithelial apoptosis, and aggravated pathological changes in the aged uterus.

Transcriptome analysis of the uterine epithelium showed that samples from the neomycin group were clearly separated from those from the control group. In total, 427 DEGs were identified (|log_2_fold change|> 1 and *P* < 0.05, Supplementary Figure S10a, b, Supplementary Table S7). We found that genes upregulated by antibiotic treatment were significantly enriched in regulating the apoptotic process and cell cycle (*P* < 0.05, Fig. 7k). In contrast, downregulated genes were enriched in the ion homeostasis and transmembrane transporter activity (*P* < 0.05, Supplementary Figure S10c). These results highlight the vulnerability of cell programming to microbial shifts in the uterus. Moreover, KEGG enrichment analysis revealed crucial regulatory targets of the uterus in response to microbial shifts (*P* < 0.05, Fig. 7l). In brief, upregulated expression of *FGF1* and *ANGPT1* can activate the mitogen-activated protein kinase (MAPK) and Phosphatidylinositol-3-kinase-Protein kinase B signaling pathways and mediate *FOS* and *SGK3* to promote cell proliferation and cell cycle progression (Supplementary Figure S10d, e). Meanwhile, *FGF1* also activates cancer-related pathways to regulate cell programming by upregulating the expression of *Wnt11*. The results indicated that antibiotic intervention induced microbiota dysbiosis, inhibition of cell apoptosis, and alteration of gene expression patterns involved in cell programming, suggesting that the interaction homeostasis was disrupted between microbiota and uterine epithelium in the host.

**Fig. 8:**
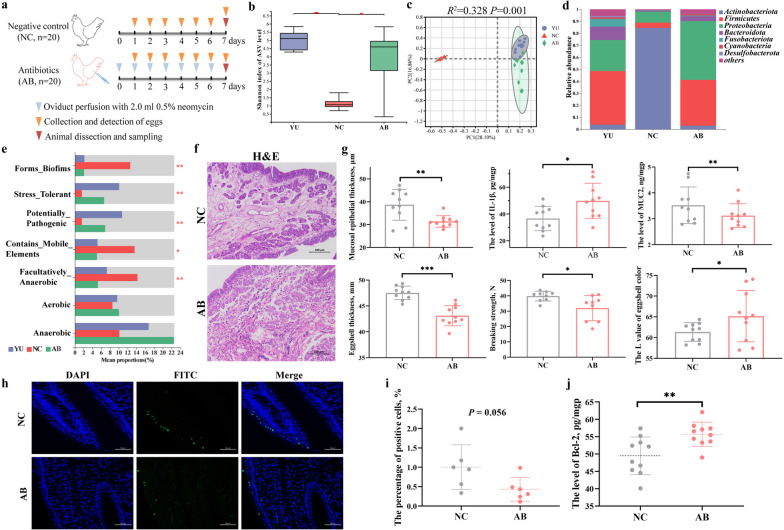
Effects of oviduct perfusion with antibiotics on uterine microbiota and microenvironment in the aged hens. **a** Experimental design overview. **b** Comparison of Shannon index of α-diversity (*n* = 10). **c** Principal coordinate analysis (PCoA) of uterine microbiota based on Bray–Curtis (*n* = 10). **d** Microbial composition at the phylum level (*n* = 10). **e** The microbial phenotype analysis based on the BugBase database (*n* = 10). **f** Effects of oviduct perfusion with antibiotics on the morphological structure of the uterus (*n* = 10). Scale bar = 100 μm. **g** Comparison of the eggshell quality and uterine microenvironment in terms of mucosal epithelial thickness, IL-1β, and MUC2 (*n* = 10). **h** Detection of cell apoptosis in uterine tissue using the TUNEL assay (*n* = 6). Scale bar = 50 μm. **i** The proportion of cell apoptosis (*n* = 6). **j** The level of B cell lymphoma-2 (Bcl-2) in the uterus mucosa (*n* = 10). NC, the negative control group; AB, the antibiotic group. IL-1β: Interleukin-1β; MUC2: Mucin-2

Furthermore, we directly interfered with the microbiota using antibiotics by uterine perfusion. To exclude the effect of perfusion treatment on uterine physiology, we first conducted oviducal perfusion with PBS (Supplementary Figure S11a). The results showed no significant differences in uterine morphology, and no significant changes were observed in mucosal epithelial thickness, IL-1β, and MUC2 levels of the aged uterus (Supplementary Figure S11b, c). In terms of eggshell quality, we found that oviducal perfusion with PBS also did not affect eggshell thickness, breaking strength, and eggshell color (Supplementary Figure S11c). Moreover, PBS did not interfere with uterine cell apoptosis, which was supported by the results of the TUNEL assay and anti-apoptotic proteins Bcl-2 (Supplementary Figure S11d–f). Consequently, the effect of oviducal infusion with neomycin on uterine physiology was investigated using PBS as a solution (Fig. 8a). Interestingly, after the infusion of neomycin in the aged oviduct, we found that the microbial composition of the aged uterus was highly similar to that of the young uterus (Fig. 8b–e). The infusion of neomycin significantly increased the microbial Shannon index and abundance of *Firmicutes* and *Proteobacteria* in the aged uterus, which was not significantly different from that in the young uterus (Fig. 8b, d). Principal coordinate analysis showed that samples from the aged uterus with perfusion were clearly clustered with young microbial samples (*R*^2^ = 0.328,* P* = 0.001, Fig. 8c). Moreover, it was more consistent in microbial phenotypes between the aged uterus with perfusion and the young uterus, especially in the form of biofilms, stress-tolerant, potentially pathogenic, containing mobile elements, and facultatively anaerobic (Fig. 8e). Consistent with the results observed in the antibiotic feeding trial, perfusion treatment with antibiotics significantly reduced mucosal epithelial thickness of the aged uterus and aggravated infiltration of lymphocytes and granulocytes, accompanied by a significantly increased level of IL-1β (Fig. 8f, g). Meanwhile, egg quality, especially eggshell quality in terms of eggshell thickness, breaking strength, and eggshell color, was significantly reduced in the aged uterus after microbial restoration (Fig. 8g). In addition, the results of the TUNEL assay showed that antibiotic infusion significantly decreased the number of apoptotic cells in the aged uterus, which was further confirmed by a higher level of anti-apoptotic protein Bcl-2 (Fig. 8h–j). These results confirm that antibiotic-induced microbial dysbiosis in the aged uterus regulates the epithelial cell cycle and apoptosis, which further implicates the important role of uterine microbiota in aged physical homeostasis.

## Discussion

Currently, there is limited knowledge of the intricate crosstalk between microbial succession characteristics of the reproductive system and host aging. In this study, by profiling the microbiota of the oviduct, intestine, egg, and surrounding environment, we first identified the effects of microbial transmission on the oviducal microbiota in hens and surprisingly found uterine microbiota of aged hens reduced microbial transmission from the lower to the upper reproductive tract. Furthermore, integrated multi-omics sheds light on the potential of aged uterine microbiota in the regulation of the host cell cycle and apoptosis through microbial metabolites involved in amino acid metabolism and biosynthesis of various secondary metabolites. Additionally, direct and indirect approaches were used to disrupt the aged uterine microbiota, contributing to verifying the role of the oviducal microbiota. In particular, we determined that microbiota dysbiosis altered cell programming and aggravated pathological changes in the aged uterus, highlighting the specificity of interactions between the uterine microbiota and the aged phenotype.

Emerging evidence suggests the transmission of intestinal microbes to the reproductive tract in poultry [36]. Anatomically, the cloaca is the end of poultry’s reproductive and digestive tracts, suggesting that the gut and oviduct microbiota may establish links by mediating the cloaca. Indeed, the current study revealed the effects of transmission patterns on microbial communities from gut to oviduct in young hens by SourceTracker analysis. In brief, microbes in the intestine can pass through the cloaca to the oviduct. Furthermore, they can successively travel through the vagina, uterus, and magnum, thereby affecting the microbial communities of the entire reproductive tract. Moreover, we found that the gut and microbes from the surroundings, such as cages, contribute to the oviduct microbial composition via the cloaca. These findings confirm that the oviducal microbiota can be shaped by horizontal transmission derived from the gut other than genetics in poultry [18]. Additionally, it may be expanded to many vertebrates with a cloaca, such as birds, reptiles, and amphibians. However, the uterine microbiota of aged hens reduced microbial transmission from the lower to the upper reproductive tract, which might be attributed to the selectivity of the reproductive tract for microbial colonization in different ecosystem niches [37]. There was potential selectivity of microbial colonization in the upper (uterus and magnum) and lower (cloaca and vagina) reproductive tracts of hens, which was also found in a previous report in humans [5]. The distinct microbial communities suggest differences in the function of the microbial ecosystem niche between the uterus and the vagina. Meanwhile, the similar microbial composition emphasizes the critical role of the uterine microbiota in microbial colonization of the magnum.

The change in the aged uterine microenvironment could be a key determiner of microbial transmission patterns, although the microbiota composition of the gut and reproductive tract evolves over age. Consistent with previous studies in humans [1], a significantly decreased Shannon index of the aged oviduct microbiota was found in the current study. Critical factors affecting microbial transmission and colonization have been confirmed, which include pH, mucins, antibacterial proteins, immunoglobulins, aerobic environment, and nutrients available in the oviduct [38]. Higher levels of MUC2 and antibacterial proteins in the uterus of aged hens compared to young hens may explain, in large part, the distinct microbial communities in the aged uterus. Therefore, regulating the uterus, especially innate immunity for bacterial colonization, obstructs the effects of microbial transmission from the lower to upper reproductive tract in aged hens.

In turn, the characteristic colonization of microbiota adapts to changes in the host microenvironment and plays a specific regulatory role [39]. Consistent with previous studies [14], we also observed enhanced innate immune defense in the uterine microenvironment of aged hens, accompanied by inflammatory pathology, especially elevated inflammatory cell infiltration and inflammatory cytokines. It is well known that microbiota dysbiosis in aged hosts can cause an elevated innate immune response and provoke chronic inflammation [40, 41]. Thus, a high proportion of *Actinobacteria* in the aged uterus relative to that in the young uterus could partially explain the enhanced innate immune defenses in the aged uterus. In addition, senescent cells are characterized by increased metabolic activity and secrete a senescence-associated secretory phenotype (SASP) dominated by inflammatory cytokines, growth factors, and proteases, triggering the inflammatory microenvironment [42]. In the current study, increased IL-1β, uterine edema, and inflammatory cell infiltration indicated that the aged uterus tends toward a pro-inflammatory microenvironment [43]. Interestingly, we found a high abundance of *Actinobacteria*, especially *Rhodococcus,* in the aged uterus. Indeed, given that metabolites produced by *Actinobacteria* have anti-inflammatory, immunomodulatory, and antibacterial activities [44], *Actinobacteria* are considered to play a key role in maintaining host microenvironment homeostasis [45]. Likewise, numerous studies have confirmed the high immunostimulatory and immunoregulatory activities of trehalolipids produced by nonpathogenic *Rhodococcus* [46]. Overall, the aged uterine microbiota is essential in regulating microenvironmental immune homeostasis.

However, innate immune-induced chronic inflammatory responses in the uterine microenvironment seem to be invariably associated with cell programming, particularly cell cycle arrest, and apoptosis. Generally, persistent inflammatory stress can induce DNA damage and further drive cell cycle arrest in senescent cells [42, 47]. Prolonged cell cycle arrest can lead to genetic mutations due to genomic instability, increasing anti-apoptotic activity, and inhibiting apoptosis, which are prerequisites for developing cancer and degenerative diseases [42, 48, 49]. Although rodents can be induced or genetically manipulated to establish reproductive tract disease models for human diseases [50], the non-spontaneous nature of these models restricts their clinical relevance. In hens, long-term and continuous egg-laying induces adverse changes and damages the oviduct tissue in the late production phase [13]. In particular, the expression of the pro-inflammatory cytokines IL-1β, IL-6, and interferon-gamma in the uterus increases during eggshell formation [43], suggesting that the oviduct of hens is persistently exposed to inflammatory stress during an ovulatory cycle. Thus, hens are the only available animal that develops ovarian cancer spontaneously with a high incidence rate, which is a suitable model for studying cancer-related diseases of the reproductive system in humans [45, 51]. The current study revealed differential patterns of uterine gene expression in aged hens, showing an altered cell cycle and inhibition of apoptosis in the aged uterus. In this light, our results implicate that uterine cell cycle arrest triggers the upregulation of the anti-apoptotic protein Bcl-2, causing decreased apoptosis, which was previously regarded as a spontaneous tumorigenesis process induced by senescence in hens [52].

As a regulator of the host immune and inflammatory responses, the commensal microbiome has the potential to affect cell apoptosis and tumorigenesis [6, 37, 53]. In long-term evolution, symbiotes and pathogens have developed various survival strategies to occupy niches in the host mucosa microenvironment [54]. Cyclomodulins, including cytotoxic necrotizing factors, DNA-damaging genotoxins, and cycle inhibitory factors, are recognized as the most direct survival strategies for bacteria [55]. As a target for cyclomodulins, the cell cycle is modulated to disrupt cell functions, contributing to optimal conditions for prolonged bacterial colonization [56]. Meanwhile, host cells acquire genomic instability after exposure to cyclomodulins, promoting malignant transformation during tumorigenesis [57]. Likewise, our results showed that the characteristic colonization of the microbiome in the uterus is closely related to senescent phenotypes in terms of cell cycle and apoptosis. In addition, emerging evidence has indicated that gut microbial roles in the development of pathological conditions are frequently mediated by bacterial metabolites, such as products of amino acid metabolism, bioamines, secondary bile acids, and short-chain fatty acids (SCFAs), which act as signaling molecules and substrates of metabolic reactions [58, 59]. The current study found that the significantly increased metabolites in the aged uterus were mainly enriched in pathways of various amino acid metabolisms, which was consistent with human aging-induced changes in gut microbial metabolites in previous studies [60]. In fact, amino acid metabolism is crucial in regulating tumorigenesis [61, 62]. Mechanistically, amino acids are essential nutrients for immune cells to maintain homeostasis in organ development and immune response. Dysregulation of amino acid consumption in immune cells due to metabolic reprogramming in the tumor microenvironment is an important cause of impaired anti-tumor immunity [63]. Meanwhile, the enhanced microbial metabolism of aromatic amino acids, including tyrosine, phenylalanine, and tryptophan, was found in the aged uterus, which previously indicated impaired permeability and immunity of the host microenvironment and colorectal carcinogenesis [64, 65]. In particular, microbial-derived indole and its derivatives through tryptophan metabolism have been confirmed to suppress anticancer immune responses and promote the malignant characteristics of cancer cells in terms of cell cycle and apoptosis [66, 67]. In addition, several biogenic amines such as tyramine, spermidine, sphingosine, phenylethanolamine, 2-hydroxyphenethylamine, tryptamines, and trimethylamine-N-oxide were also increased in the aged uterus, which also occurred in patients with bacterial vaginosis and was related to the activation of immune and inflammatory responses [4]. In general, our results demonstrate that the uterine microbiome and its metabolites mediate key senescent features in terms of the cell cycle and apoptosis in aged hens, providing a mechanistic basis for the targeted modulation of microbial-host interactions.

Up to now, numerous aging-related studies have focused on reshaping the aged microbiome to improve the host physiological microenvironment [68]. However, emerging evidence indicates that replacing the microbiota with a young pattern may not be the optimal approach [69, 70]. Consistently, our results also confirmed that the disruption of the uterine microbiota did not improve aging-related physiological changes but further aggravated aging processes by disrupting cell programming involved in the cell cycle, proliferation, and apoptosis. Meanwhile, the eggshell quality of aged hens was worse after the antibiotic intervention, which further demonstrated weakened physiological functions of the uterus. We speculate that the balance in immunological tolerance between the microbiome and microenvironment of the aged uterus has been disrupted, causing disturbed immune defenses in the uterus and aggravating aging changes [71]. Moreover, increasing uniqueness (Bray–Curtis distance) in the individual gut microbiome was observed with age, which was positively correlated with known microbial metabolic markers involved in immune regulation, inflammation, aging, and longevity [60]. Particularly, some beneficial microbial metabolites increase with the healthy aging of the host. However, they may not be readily synthesized in the young microbiome [69]. In the current study, increased production of SCFAs, including butyric acid, 2-hydroxy-3-methyl butyric acid, mesaconic acid, isovaleric acid, 2-hydroxypentanoic acid, and 6-hydroxyhexanoic acid, was found in the aged uterine microbiome. Previous studies have demonstrated that SCFAs play an anticancer role by regulating immune and inflammatory responses and cell cycle [58], suggesting an improved role in aging-related physiological changes. Likewise, benzofuran has various biological activities as the most distinctive microbial metabolite in the aged uterus, especially anti-inflammatory and anti-tumor [72], emphasizing the positive effects on the cell cycle and apoptosis in the aged uterus. Overall, the dual roles of microbial metabolites in promoting and preventing aging-related physiological changes implicate the adaptation of microbiota to the aged uterus [60, 69, 73]. Therefore, future research needs to focus on the role of microbes in specific microenvironments to apply a more tailored strategy for improving aging-related physiological changes.

## Conclusions

The microbiota continuum along the reproductive tract in aged birds differs from that in young birds, especially with a significant shift in the uterus. Aged uterine microbiota is an adaptive colonization of the senescent microenvironment and probably regulates cell programming in terms of cell cycle and apoptosis. In particular, the inhibition of apoptosis by disrupting immune homeostasis and the cell cycle is associated with microbial metabolites primarily involved in amino acid metabolism and the biosynthesis of various secondary metabolites. Uterine microbial dysbiosis caused by antibiotic intervention aggravates age-related physiological changes in aged hens. Our study is the first to demonstrate that the microbiota contributes to cellular and physiological changes in the aged uterus. Furthermore, it highlights the interaction between microbiota shifts and uterine immune defense homeostasis during aging. These findings provide new insights into the roles of the reproductive tract microbiota in regulating the cell programming of the aged host, contributing to the exploration of the microbiome as a target for diagnosing health status and possible therapy for the aged reproductive tract.

### Supplementary Information

**Additional file 1: Figure S1.** A relative abundance bar graph of microbial composition for all samples.

**Additional file 2: Figure S2.** Spearman’s correlation analysis between microbiota and changes in uterine microenvironment. ET, the epithelial thickness of mucosa; MUC2: Mucin-2; IL-1β: Interleukin-1β; IgA: Immunoglobulin A; IgG: Immunoglobulin G; IgM: Immunoglobulin M; OVT, ovotransferrin; LZM, lysozyme.

**Additional file 3: Figure S3.** Analysis of the transcriptome and PPI network based on the STRING database. c-g, Genes of clusters 1-5, respectively.

**Additional file 4: Figure S4.** RT-PCR validation of key differential genes in the transcriptome.

**Additional file 5: Figure S5.** The metabolomic analyses. a Principal component analysis. b Volcano plot of significantly differential metabolites (VIP >1 and* P* < 0.05). c Pathway enrichment analysis of downregulated metabolites in aged hens.

**Additional file 6: Figure S6.** O2PLS model with 10-fold cross-validation for metabolomics and transcriptomics.

**Additional file 7: Figure S7.** Metabolic overview map annotated by the differential metabolite and differential gene sets.

**Additional file 8: Figure S8.** Effects of dietary antibiotic supplementation on cecal and uterine microbiota. a-b Differential bacteria of the cecal and uterine microbiota.

**Additional file 9: Figure S9.** Effects of dietary antibiotic supplementation on eggshell quality and egg production.

**Additional file 10: Figure S10.** Effects of dietary antibiotic supplementation on the uterine transcriptome. a Principal component analysis of the transcriptome. b Volcano plot of differentially expressed genes. c Significantly enriched Gene Ontology (GO) terms of downregulated genes in the antibiotic group. d The crucial regulatory targets of the uterus in response to microbial shifts in aged hens. RT-PCR validation of key regulatory genes in the transcriptome.

**Additional file 11: Figure S11.** Effects of oviduct perfusion with PBS on uterine microenvironment in the aged hens. a Experimental design overview. b Effects of oviduct perfusion with PBS on the morphological structure of the uterus. Scale bar = 100 μm. c Comparison of the uterine microenvironment and eggshell quality. d Detection of cell apoptosis in uterine tissue using the TUNEL assay. Scale bar = 50 μm. e The proportion of cell apoptosis. f The level of B-cell lymphoma-2 (Bcl-2) in the uterus mucosa. NC, the negative control group; PBS, the PBS perfusion group. IL-1β: Interleukin-1β; MUC2: Mucin-2.

**Additional file 12: Table S1.** Composition and nutrient levels of the basal diet.

**Additional file 13: Table S2.** Primer sequences of target and reference genes used for RT-PCR in this study.

**Additional file 14:** **Table S3.** The individual analysis of each two groups using PERMANOVA.

**Additional file 15:** **Table S4.** Genes differentially expressed in the transcriptome of young and aged hens.

**Additional file 16: Table S5.** Differential metabolites of the metabolome.

**Additional file 17:** **Table S6.** Spearman’s correlation analysis between differential metabolites and gene sets.

**Additional file 18: Table S7.** Differentially expressed genes in the transcriptome in the dietary antibiotic supplementation experiment.

## Data Availability

Sequencing data of 16S rDNA and transcriptomics have been deposited into the NCBI Sequence Read Archive (SRA) under the accession numbers PRJNA909442 and PRJNA909207, respectively.

## Abbreviations

ASVs: Amplicon sequence variants
Bcl-2: B cell lymphoma-2
DEGs: Differentially expressed genes
DNA: Deoxyribonucleic acid
FDR: False discovery rate
IL-1β: Interleukin-1β
IL-6: Interleukin-6
IgA: Immunoglobulin A
IgG: Immunoglobulin G
IgM: Immunoglobulin M
GO: Gene Ontology
KEGG: Kyoto Encyclopedia of Genes and Genomes
MAPK: Mitogen-activated protein kinase
MUC2: Mucin-2
PBS: Phosphate-buffered saline
O2PLS: Two-way Orthogonal PLS
PCA: Principal component analysis
PCoA: Principal coordinate analysis
PCR: Polymerase chain reaction
PERMANOVA: Permutational multivariate analysis of variance
RT-PCR: Real-time PCR
SASP: Senescence-associated secretory phenotype
SCFAs: Short-chain fatty acids
TUNEL: Terminal deoxynucleotidyl transferase-mediated dUTP nick end labeling
UHPLC-Q: Ultra-high performance liquid chromatography-quadrupole
VIP: Variable importance in the projection
