# Supplementary figures and images for: Uterine microbial communities and their potential role in the regulation of epithelium cell cycle and apoptosis in aged hens
Source: Microbiome. 2023 Nov 11;11:251. doi: 10.1186/s40168-023-01707-7 (PMC10638742; doi:10.1186/s40168-023-01707-7)

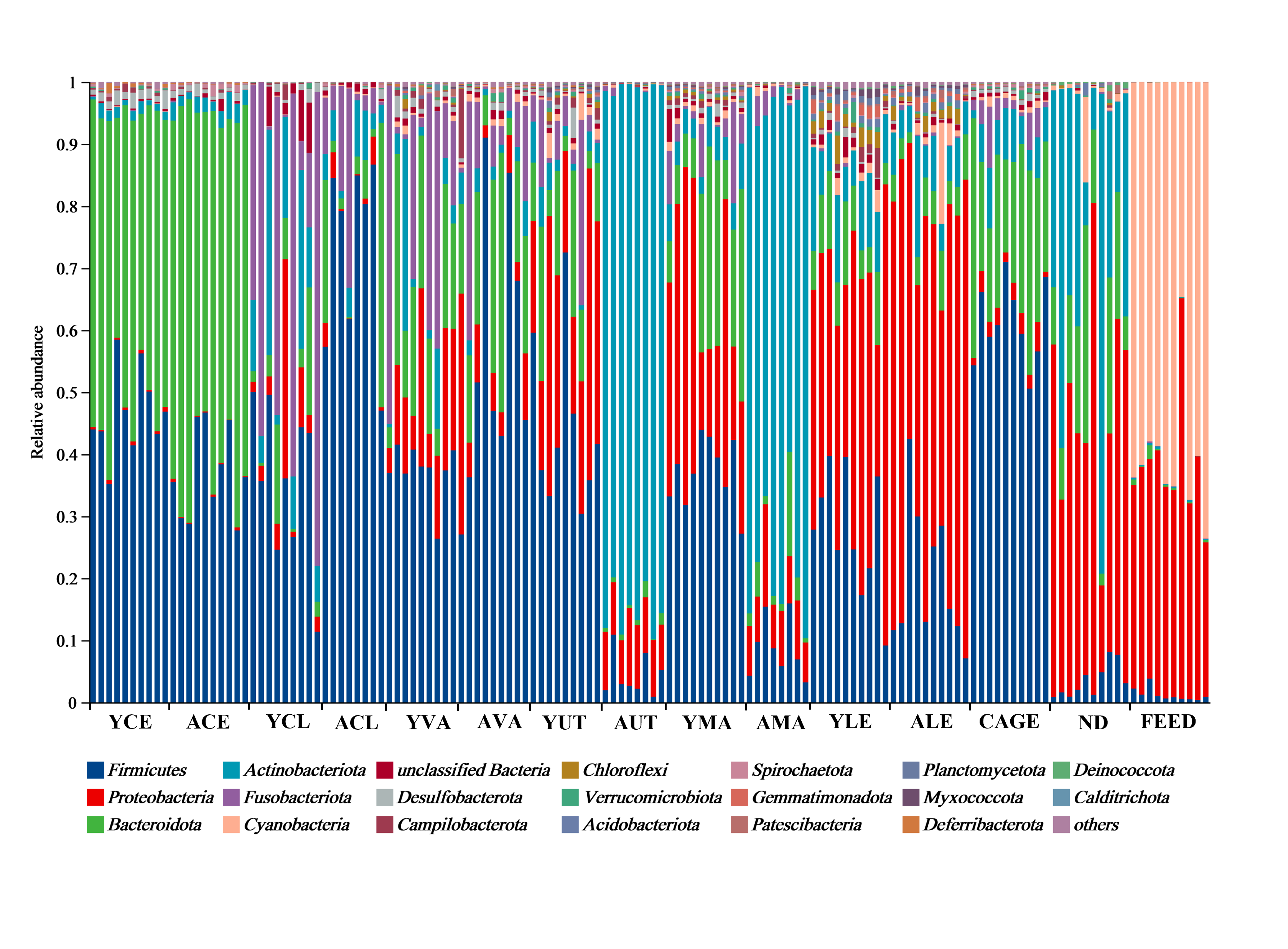

Supplement: Supplementary file 2 — Additional file 1: Figure S1. A relative abundance bar graph of microbial composition for all samples. [file 40168_2023_1707_MOESM1_ESM.tif]

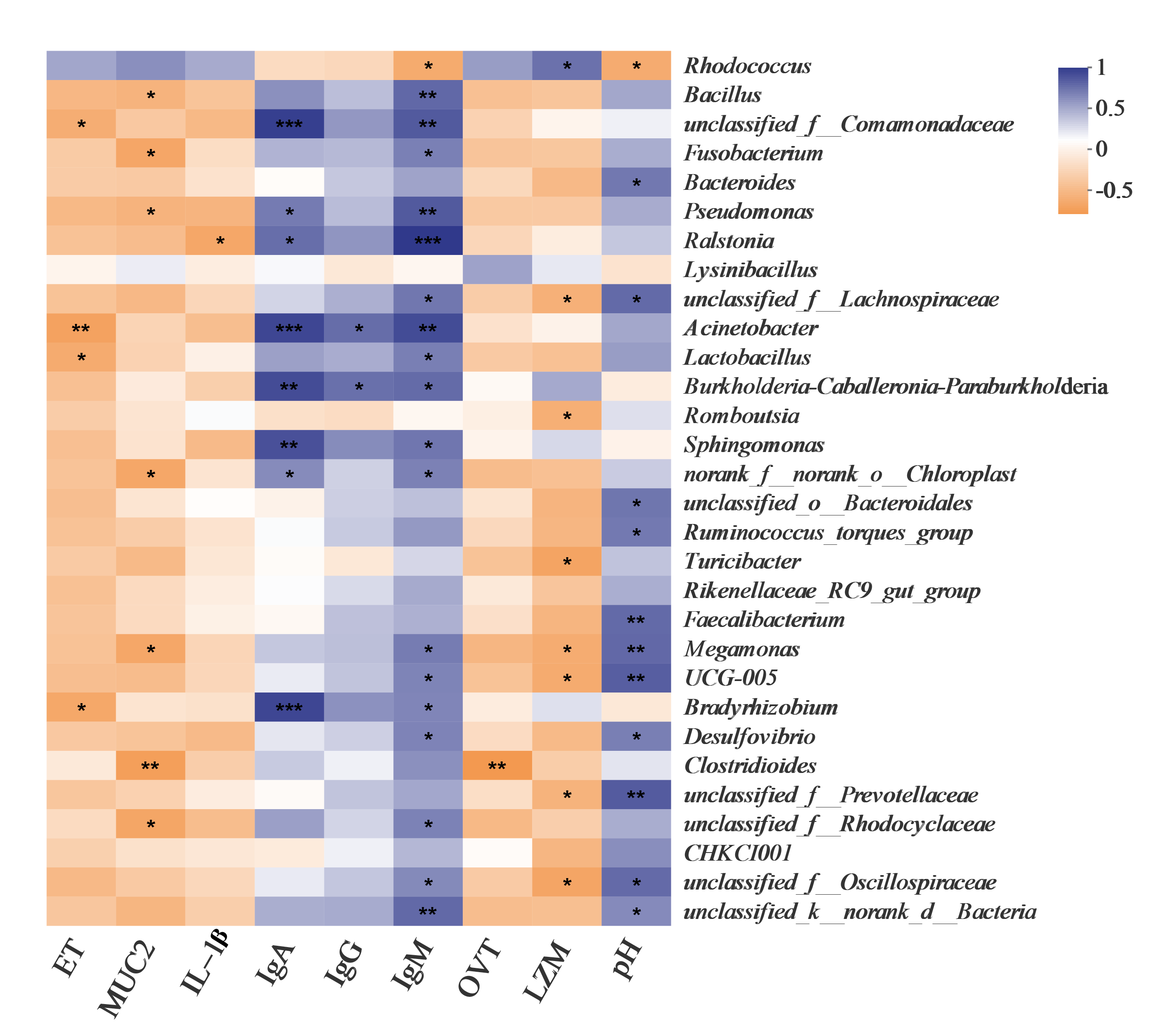

Supplement: Supplementary file 3 — Additional file 2: Figure S2. Spearman’s correlation analysis between microbiota and changes in uterine microenvironment. ET, the epithelial thickness of mucosa; MUC2: Mucin-2; IL-1β: Interleukin-1β; IgA: Immunoglobulin A; IgG: Immunoglobulin G; IgM: Immunoglobulin M; OVT, ovotransferrin; LZM, lysozyme. [file 40168_2023_1707_MOESM2_ESM.tif]

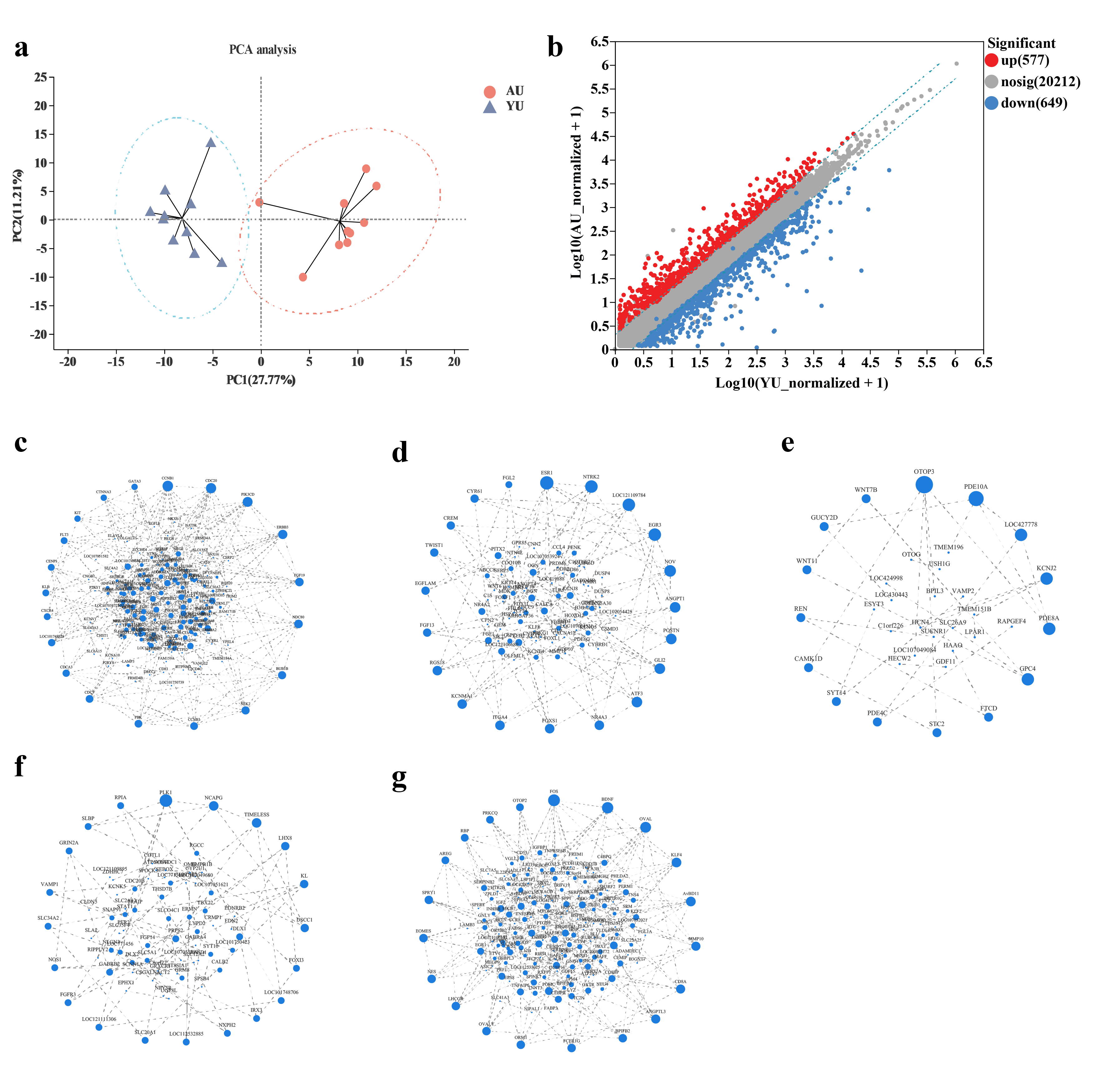

Supplement: Supplementary file 4 — Additional file 3: Figure S3. Analysis of the transcriptome and PPI network based on the STRING database. c-g, Genes of clusters 1-5, respectively. [file 40168_2023_1707_MOESM3_ESM.tif]

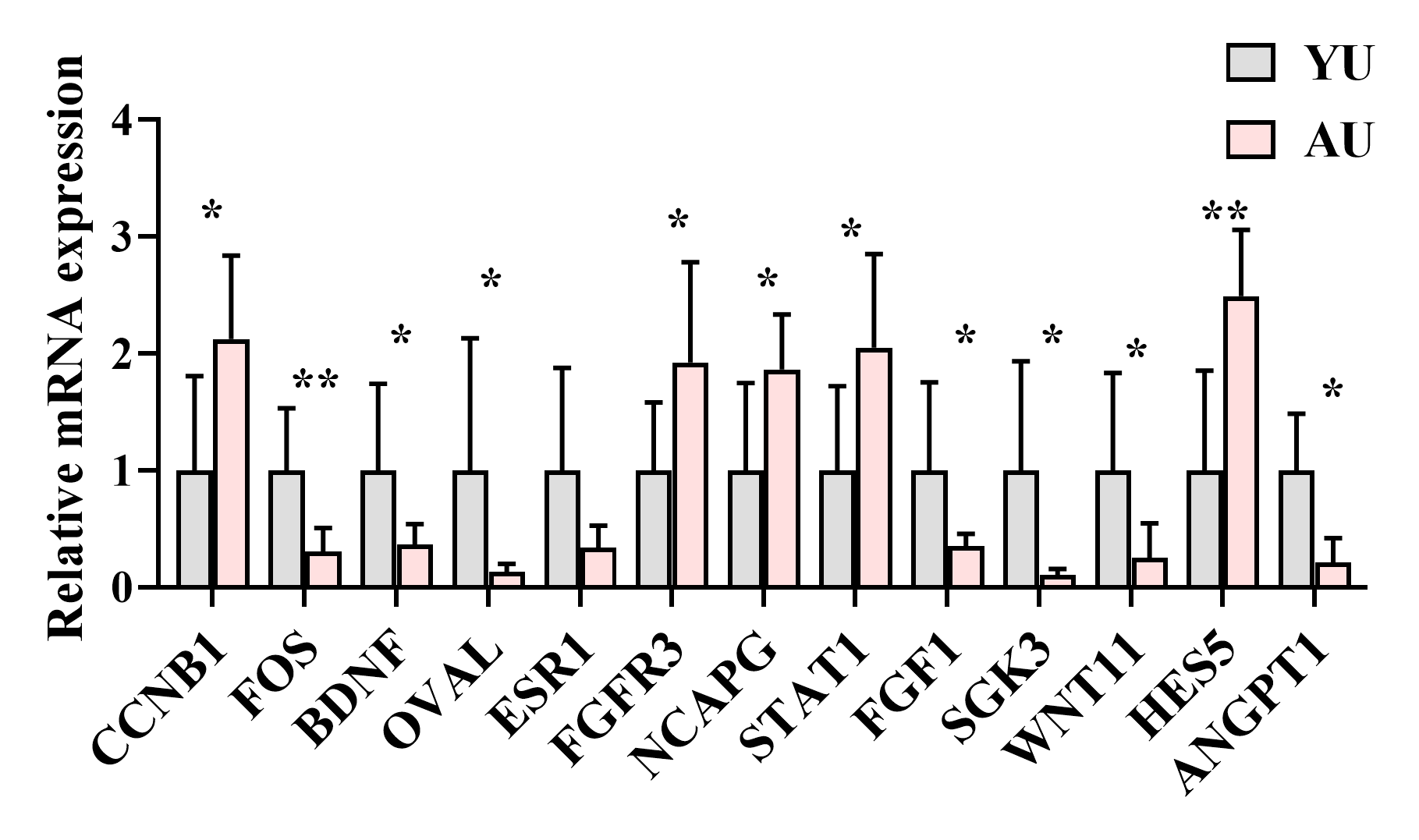

Supplement: Supplementary file 5 — Additional file 4: Figure S4. RT-PCR validation of key differential genes in the transcriptome. [file 40168_2023_1707_MOESM4_ESM.tif]

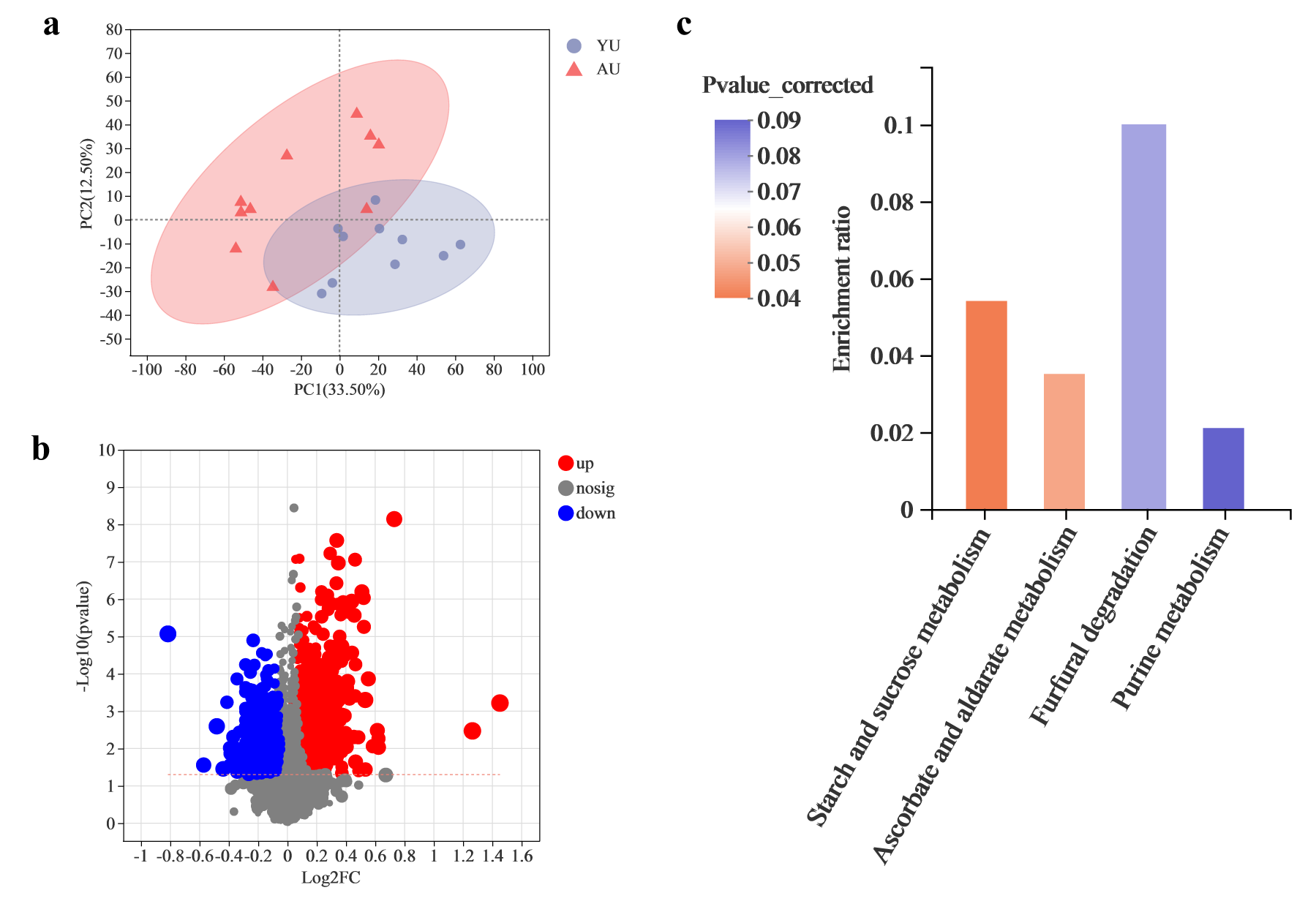

Supplement: Supplementary file 6 — Additional file 5: Figure S5. The metabolomic analyses. a Principal component analysis. b Volcano plot of significantly differential metabolites (VIP >1 and P < 0.05). c Pathway enrichment analysis of downregulated metabolites in aged hens. [file 40168_2023_1707_MOESM5_ESM.tif]

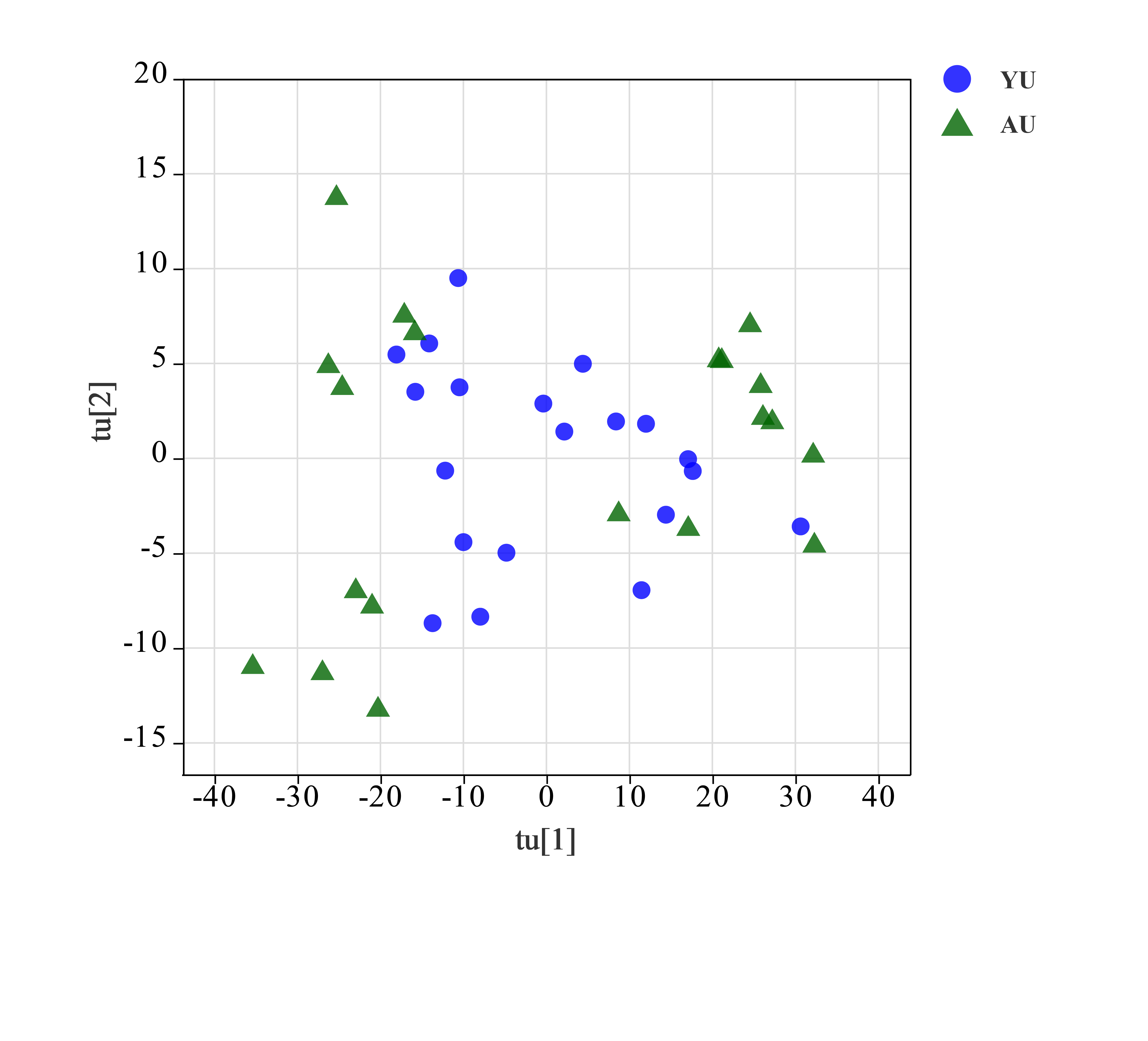

Supplement: Supplementary file 7 — Additional file 6: Figure S6. O2PLS model with 10-fold cross-validation for metabolomics and transcriptomics. [file 40168_2023_1707_MOESM6_ESM.tif]

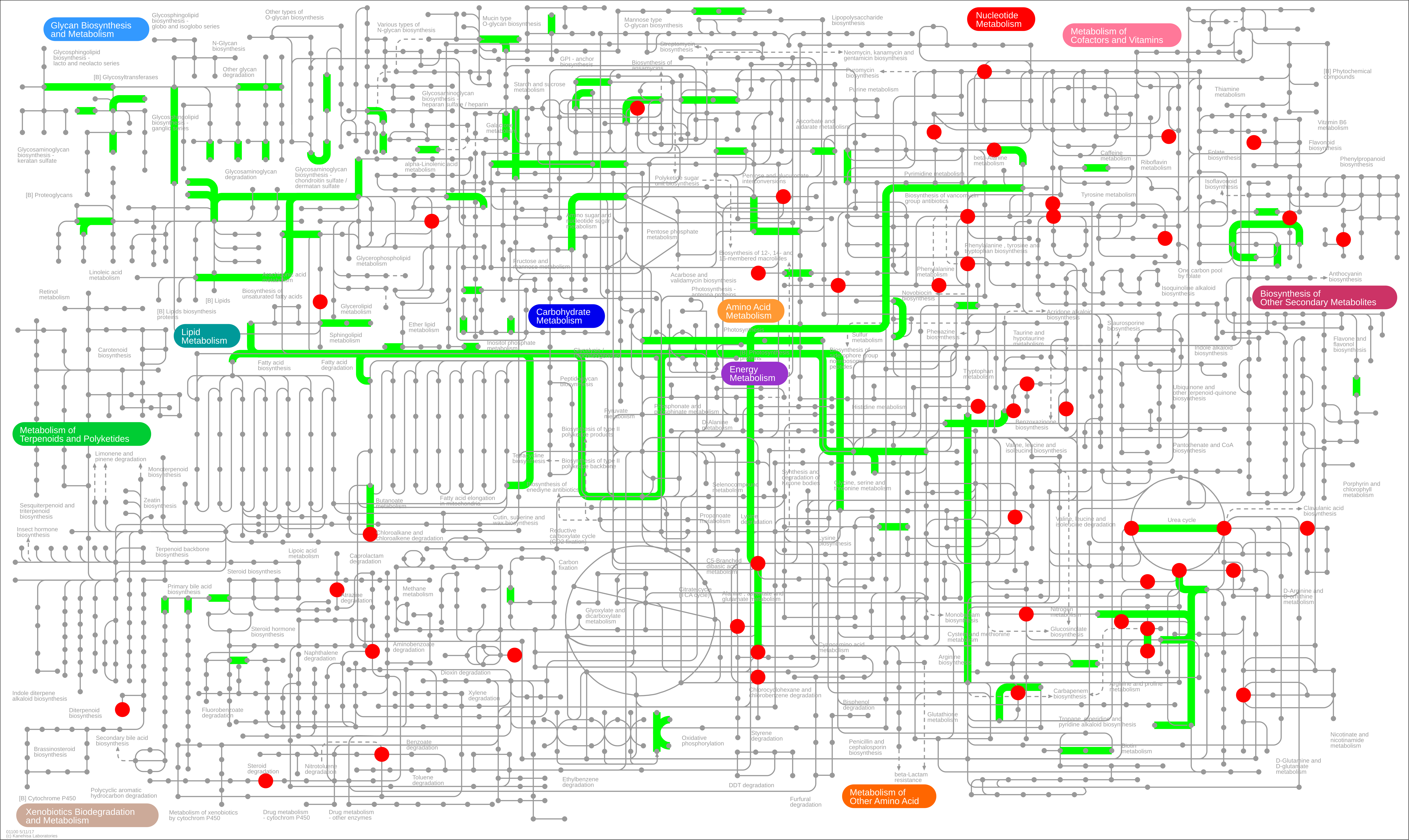

Supplement: Supplementary file 8 — Additional file 7: Figure S7. Metabolic overview map annotated by the differential metabolite and differential gene sets. [file 40168_2023_1707_MOESM7_ESM.tif]

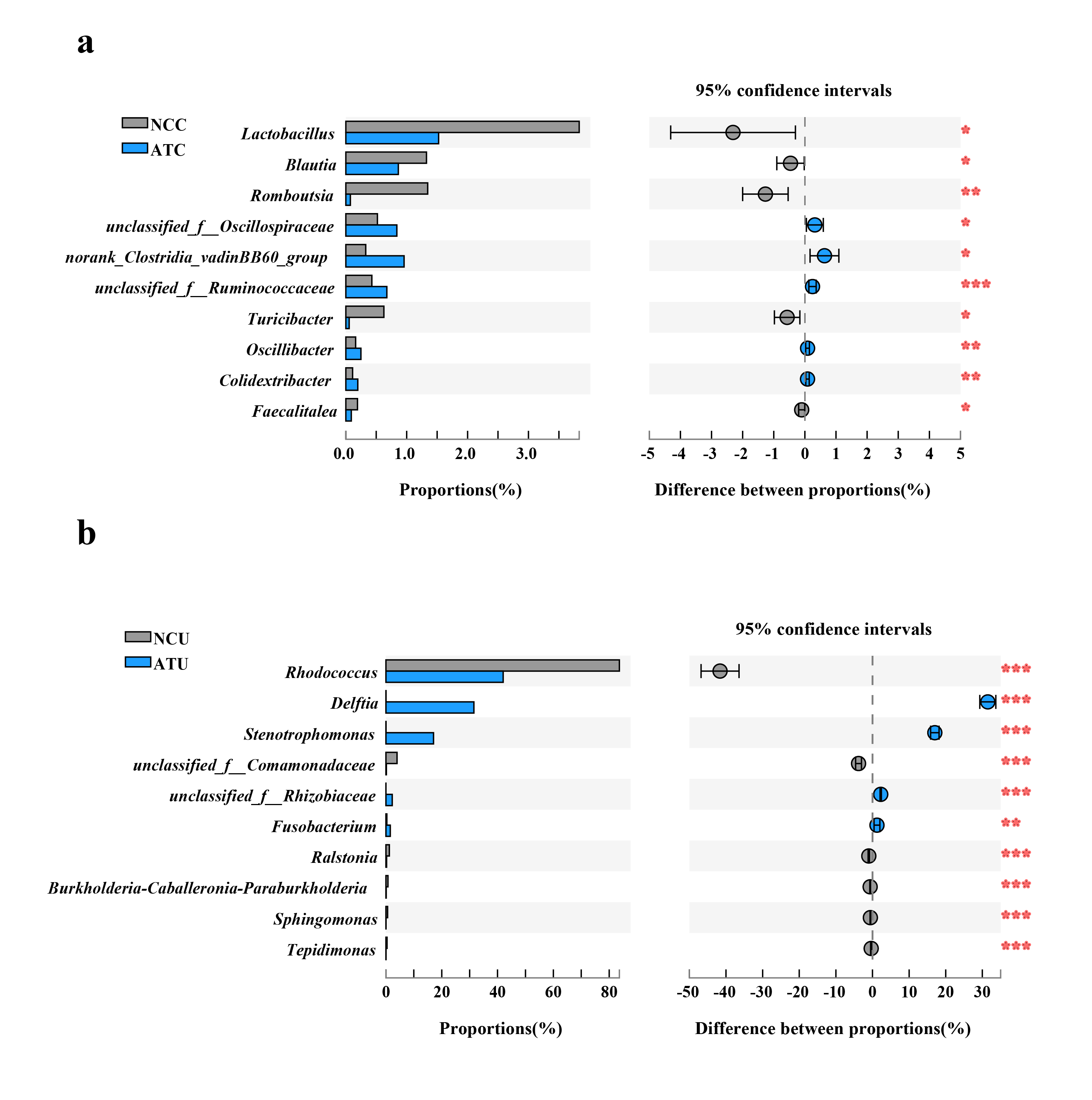

Supplement: Supplementary file 9 — Additional file 8: Figure S8. Effects of dietary antibiotic supplementation on cecal and uterine microbiota. a-b Differential bacteria of the cecal and uterine microbiota. [file 40168_2023_1707_MOESM8_ESM.tif]

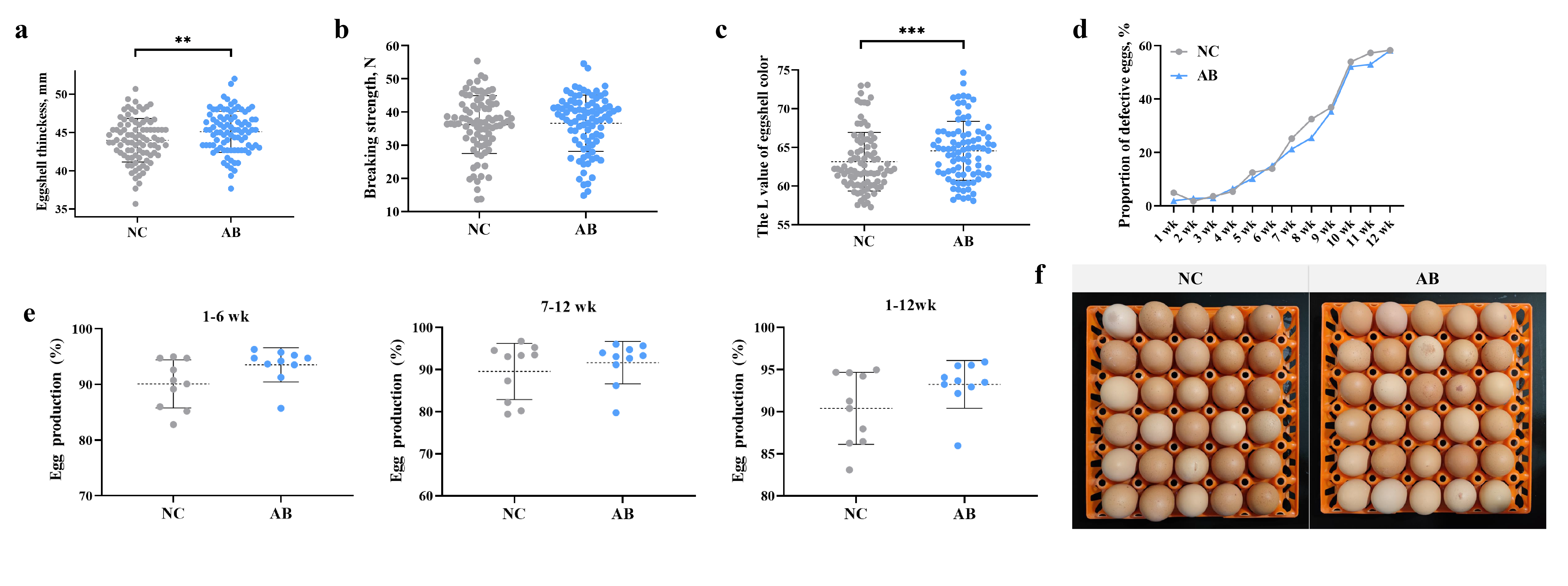

Supplement: Supplementary file 10 — Additional file 9: Figure S9. Effects of dietary antibiotic supplementation on eggshell quality and egg production. [file 40168_2023_1707_MOESM9_ESM.tif]

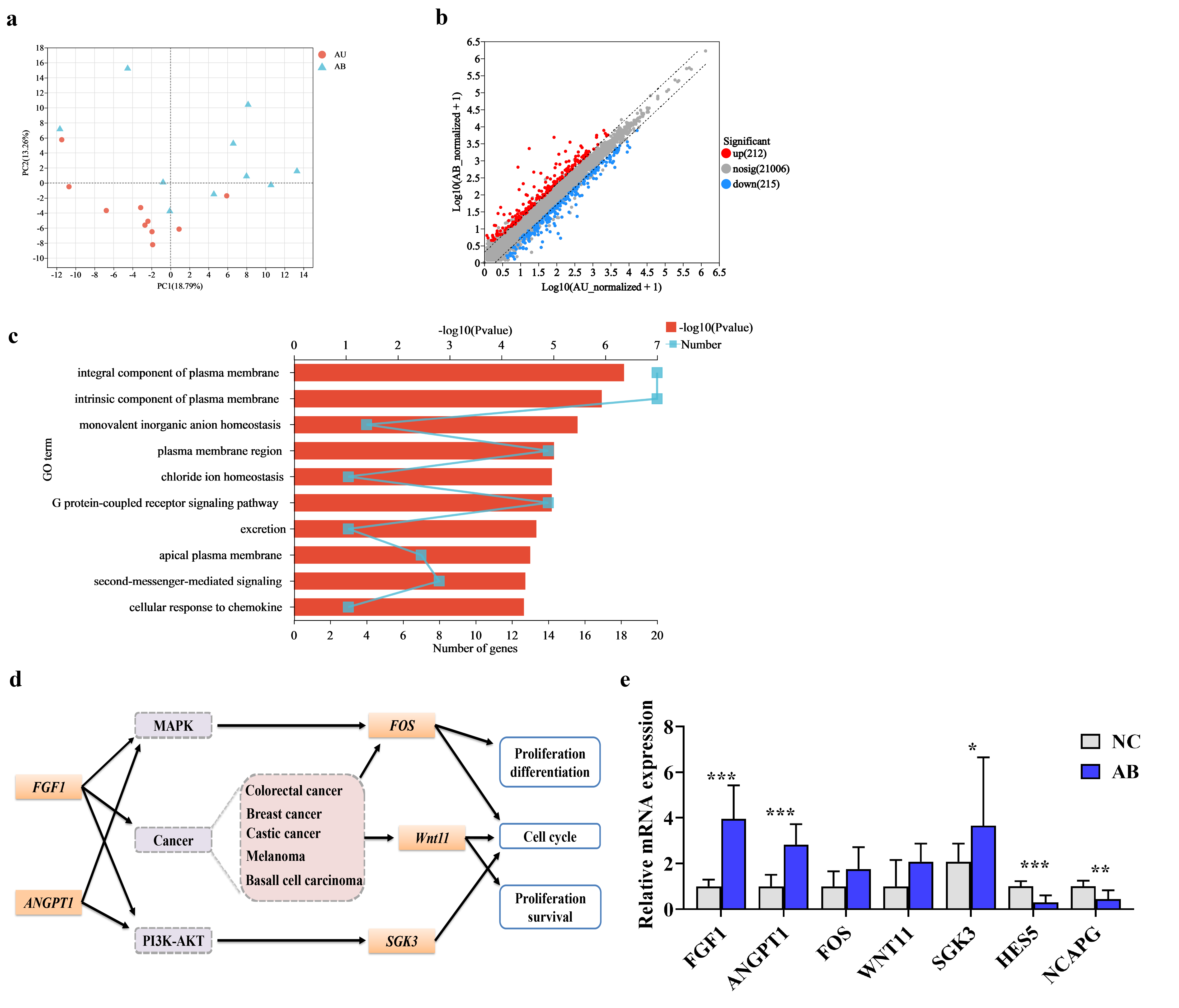

Supplement: Supplementary file 11 — Additional file 10: Figure S10. Effects of dietary antibiotic supplementation on the uterine transcriptome. a Principal component analysis of the transcriptome. b Volcano plot of differentially expressed genes. c Significantly enriched Gene Ontology (GO) terms of downregulated genes in the antibiotic group. d The crucial regulatory targets of the uterus in response to microbial shifts in aged hens. RT-PCR validation of key regulatory genes in the transcriptome. [file 40168_2023_1707_MOESM10_ESM.tif]

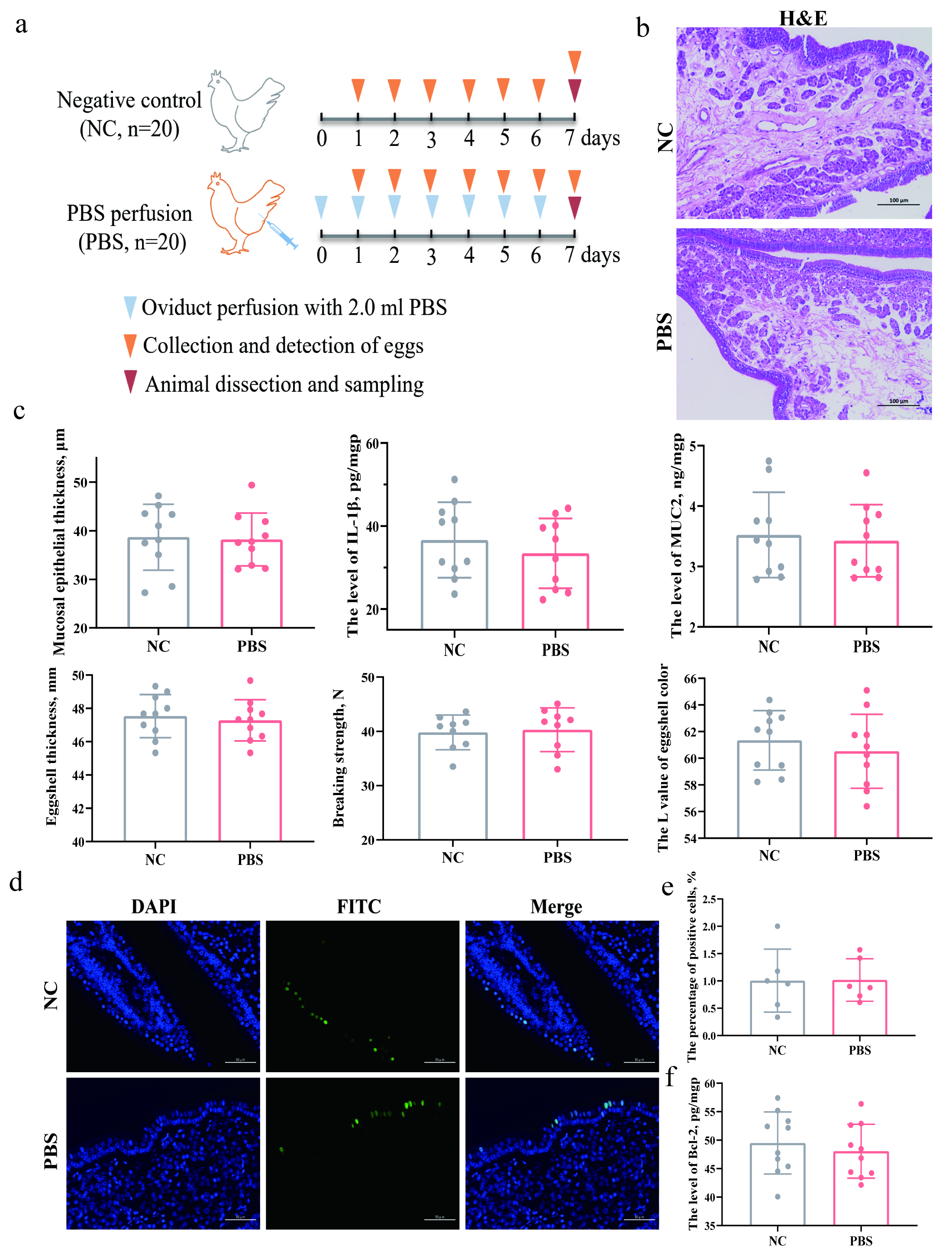

Supplement: Supplementary file 12 — Additional file 11: Figure S11. Effects of oviduct perfusion with PBS on uterine microenvironment in the aged hens. a Experimental design overview. b Effects of oviduct perfusion with PBS on the morphological structure of the uterus. Scale bar = 100 μm. c Comparison of the uterine microenvironment and eggshell quality. d Detection of cell apoptosis in uterine tissue using the TUNEL assay. Scale bar = 50 μm. e The proportion of cell apoptosis. f The level of B-cell lymphoma-2 (Bcl-2) in the uterus mucosa. NC, the negative control group; PBS, the PBS perfusion group. IL-1β: Interleukin-1β; MUC2: Mucin-2. [file 40168_2023_1707_MOESM11_ESM.tif]
