# Supplementary material for: Uterine microbial communities and their potential role in the regulation of epithelium cell cycle and apoptosis in aged hens
Source: Microbiome. 2023 Nov 11;11:251. doi: 10.1186/s40168-023-01707-7 (PMC10638742; doi:10.1186/s40168-023-01707-7)
Supplement: Supplementary file 13 — Additional file 12: Table S1. Composition and nutrient levels of the basal diet. [file 40168_2023_1707_MOESM12_ESM.docx]

**Table S1** The composition and nutrient levels of the basal diet (air dry basis)

| Ingredients | % | Nutrient levels^2^ | % |
| --- | --- | --- | --- |
| Corn | 61.00 | Metabolizable energy, MJ/kg | 11.27 |
| Soybean meal | 23.86 | Crude protein | 16.50 |
| Soybean oil | 1.20 | Calcium | 3.47 |
| Wheat bran | 3.35 | Available phosphorus | 0.27 |
| Salt | 0.15 | Lysine | 0.81 |
| Na_2_SO_4_ | 0.20 | Methionine | 0.37 |
| CaHPO_4_ | 0.90 | Methionine+cystine | 0.65 |
| Limestone | 8.90 |  |  |
| Premix^1^ | 0.20 |  |  |
| Choline | 0.12 |  |  |
| DL-Methionine | 0.12 |  |  |
| Total | 100.00 |  |  |

^1^ Provided per kilogram of diet: VA 12,500 IU; VD_3_ 4,125 IU; VE 15 IU; VK 2 mg; thiamine 1 mg; riboflavin 8.5 mg; calcium pantothenate 50 mg; niacin 32.5 mg; pyridoxine 8 mg; biotin 2 mg; folic acid 5 mg; VB_12_ 5 mg; Zn (ZnSO_4_·H_2_O) 66 mg; I (KI) 1 mg; Fe (FeSO_4_·H_2_O) 60 mg; Cu (CuSO_4_·5H_2_O) 8 mg; Se (Na_2_SeO_3_) 0.3 mg.

^2^ The nutrient levels were calculated values.
